# Longitudinal maternal hemodynamics in high‐risk pregnancies with different subtypes of pre‐eclampsia

**DOI:** 10.1002/uog.70215

**Published:** 2026-04-10

**Authors:** J. Lin, X. Wang, Y. Chen, L. Nguyen‐Hoang, A. S. T. Tai, I. S. Wong, X. Y. Chen, M. H. Ng, H. H. Y. Leung, A. P. W. Lee, L. C. Poon

**Affiliations:** ^1^ Department of Obstetrics and Gynaecology The Chinese University of Hong Kong Hong Kong SAR China; ^2^ Department of Obstetrics and Gynecology, Nanchong Central Hospital The Second Clinical Medical College of North Sichuan Medical University Sichuan China; ^3^ Fetal Medicine Centre, Tam Anh HCMC General Hospital Ho Chi Minh City Vietnam; ^4^ School of Nursing Tung Wah College Hong Kong SAR China; ^5^ Department of Medicine and Therapeutics, Prince of Wales Hospital The Chinese University of Hong Kong Hong Kong SAR China; ^6^ Shenzhen Research Institute The Chinese University of Hong Kong Hong Kong SAR China

**Keywords:** first‐trimester combined test, longitudinal analysis, maternal hemodynamics, preterm pre‐eclampsia, term pre‐eclampsia, transthoracic echocardiography

## Abstract

**Objective:**

To evaluate maternal cardiac adaptation during pregnancy by comparing longitudinal hemodynamic profiles between women identified as high risk for preterm pre‐eclampsia (PE) who subsequently developed preterm PE, term PE or remained unaffected, as well as low‐risk women.

**Methods:**

This was a prospective longitudinal study of 1078 Chinese women with a singleton pregnancy who were enrolled in a first‐trimester screen‐and‐prevent program for preterm PE at the Prince of Wales Hospital, Hong Kong SAR, China, between January 2020 and June 2024. Participants were classified as high or low risk for preterm PE based on the Fetal Medicine Foundation first‐trimester combined test. Participants were followed up at 12 + 0 to 15 + 6 weeks' gestation, 20 + 0 to 24 + 6 weeks and 30 + 0 to 37 + 6 weeks to measure hemodynamic variables, including heart rate (HR), stroke volume (SV), cardiac output (CO), systemic vascular resistance (SVR) and mean arterial pressure (MAP). Participants were categorized into four groups according to risk status for preterm PE and subsequent development of PE: low risk who did not develop PE (Group 1, *n* = 407); high risk who did not develop PE (Group 2, *n* = 598); high risk who developed term PE (Group 3, *n* = 29); and high risk who developed preterm PE (Group 4, *n* = 44). Hemodynamic variables were log_10_ transformed and analyzed using linear mixed‐effects models, adjusting for maternal characteristics and clinical visits. Estimated marginal means of log_10_‐transformed values for hemodynamic variables were compared between the groups throughout pregnancy and at each clinical visit.

**Results:**

Compared with Group 1, all high‐risk groups exhibited lower SV and CO and higher SVR and MAP throughout pregnancy (all *P* < 0.05), while HR was significantly elevated in Group 2 (*P* < 0.001). Compared with Group 2, Group 4 had lower HR, SV and CO and higher SVR and MAP throughout pregnancy (all *P* < 0.01). In contrast, Group 3 showed no significant differences in HR, SV, CO and SVR compared with Group 2 (all *P* > 0.05), although there was an increase in MAP from midgestation onwards (*P* < 0.001). Compared with Group 3, Group 4 exhibited lower CO and higher SVR and MAP throughout pregnancy (all *P* < 0.05).

**Conclusions:**

Compared with low‐risk women who do not develop PE, high‐risk women with or without preterm or term PE exhibit distinct cardiac maladaptation profiles from early to late gestation. These findings offer insight into the different pathophysiological mechanisms underlying PE subtypes and should inform risk stratification. © 2026 The Author(s). *Ultrasound in Obstetrics & Gynecology* published by John Wiley & Sons Ltd on behalf of International Society of Ultrasound in Obstetrics and Gynecology.

## INTRODUCTION

Pre‐eclampsia (PE), which affects 2–5% of pregnancies, is a multisystem disorder of pregnancy that contributes substantially to maternal and neonatal morbidity and mortality worldwide^1^, ^2^, ^3^. It is a clinically heterogeneous disorder, with preterm PE (delivery < 37 weeks' gestation) associated typically with more severe adverse outcome compared to term PE (delivery ≥ 37 weeks' gestation)^4^, ^5^, ^6^. To identify women at elevated risk for preterm PE in early pregnancy, the Fetal Medicine Foundation (FMF) developed a widely adopted test incorporating maternal demographic characteristics, mean arterial pressure (MAP), uterine artery (UtA) Doppler pulsatility index (PI) and serum placental growth factor (PlGF), which has been shown to identify 65–75% of preterm PE cases at a 10% false‐positive rate^7^, ^8^, ^9^, ^10^. While this screening tool has improved early prediction of PE, the pathophysiological mechanisms underlying different subtypes of PE remain unclear.

It is increasingly recognized that PE reflects inadequate maternal cardiovascular adaptation to pregnancy^11^, ^12^, ^13^, ^14^, ^15^, ^16^. However, studies have variably characterized PE as a hyperdynamic, hypodynamic or normodynamic condition, reflecting inconsistencies in the literature^17^, ^18^, ^19^, ^20^, ^21^, ^22^, ^23^, ^24^. Some studies have suggested two types of maternal circulation during PE: low cardiac output (CO)/high total vascular resistance (TVR) in early PE (delivery < 34 weeks' gestation) and high CO/low TVR in late PE (delivery ≥ 34 weeks' gestation)^17^, ^18^, ^19^. Others have reported that PE with fetal growth restriction is associated with a hypodynamic profile, while PE without fetal growth restriction may have a normodynamic or hyperdynamic status^15^, ^20^, ^21^, ^22^, ^23^, ^24^. Much of the evidence is based on cross‐sectional data^15^, ^16^, ^17^, ^20^, ^21^, ^23^, ^24^, ^25^, small cohorts^13^, ^20^, ^22^, ^26^ and/or at‐risk pregnancies^13^, ^15^, ^17^, and lacks adequate adjustment for maternal characteristics^13^, ^15^, ^17^, ^20^, ^21^, ^22^, ^23^, ^26^, ^27^. While a few longitudinal studies exist, they have not investigated specifically whether the subtypes of PE (preterm *vs* term) are associated with distinct patterns of hemodynamic adaptation^13^, ^26^, ^27^, ^28^, ^29^, ^30^. To date, no longitudinal study has evaluated maternal hemodynamic changes throughout gestation in relation to both first‐trimester FMF‐based risk stratification and subsequent development of preterm or term PE.

Therefore, this study aimed to evaluate maternal cardiovascular adaptation during pregnancy by comparing longitudinal hemodynamic profiles among women identified as high‐risk for preterm PE who subsequently developed preterm PE, high‐risk women who developed term PE, high‐risk women who remained unaffected and low‐risk women who remained unaffected.

## METHODS

### Study design and population

This was a prospective longitudinal study, extending the cohort described previously by Wang *et al*.^16^, ^31^, involving women with a singleton pregnancy who participated in the first‐trimester screen‐and‐prevent program for preterm PE at the Prince of Wales Hospital, Hong Kong SAR, China, between January 2020 and June 2024. According to the first‐trimester FMF combined test, participants were identified as being at high risk (adjusted risk ≥ 1:100) or low risk (adjusted risk < 1:100) for preterm PE. High‐risk women weighing < 40 kg were offered aspirin prophylaxis at a daily dose of 100 mg, while those weighing ≥ 40 kg were offered aspirin prophylaxis at a daily dose of 160 mg, starting before 16 weeks until 36 weeks, or until delivery or the onset of PE before 36 weeks^10^, ^32^, ^33^, ^34^. To ensure comparability between the study groups, low‐risk women were matched 1:1 with recruited high‐risk women based on maternal age (within ± 3 years), weight (within ± 5 kg) and date of screening (within ± 14 days).

The inclusion criteria were maternal age ≥ 18 years, singleton pregnancy with a viable fetus at 11 + 0 to 13 + 6 weeks' gestation and Chinese ethnicity. Exclusion criteria included: multiple pregnancy; major fetal abnormality detected on first‐trimester ultrasound; pre‐existing maternal cardiac disease, with the exception of chronic hypertension (CH); inability to provide written informed consent or learning difficulties; pregnancy ending in termination or miscarriage < 24 weeks' gestation; fewer than two clinical visits during the study period; and low‐risk women who developed PE. Written informed consent was obtained from all participants in the present study and ethical approval was granted by the Joint Chinese University of Hong Kong–New Territories East Cluster Clinical Research Ethics Committee (CREC reference number: 2016.292).

### Maternal characteristics and pregnancy outcome

Gestational age was determined based on fetal crown–rump length measured during the first trimester^35^. Maternal characteristics, medical and obstetric history, and any history of drug use were recorded^36^. Pregnancy outcomes were obtained from the hospital's computerized maternity records or from the women's obstetricians and were recorded in our secure database. We contacted both the participants and their obstetricians to confirm the use of aspirin and to collect data on pregnancy outcomes for those who received pregnancy care and delivered outside of the Prince of Wales Hospital. PE was defined according to the guidelines of the International Society for the Study of Hypertension in Pregnancy (ISSHP)^37^. Preterm PE refers to PE resulting in delivery < 37 weeks' gestation, while term PE refers to PE resulting in delivery ≥ 37 weeks. We classified the study population into four groups based on risk status for preterm PE and subsequent development of PE: Group 1, low‐risk women who did not develop PE; Group 2, high‐risk women who did not develop PE; Group 3, high‐risk women who developed term PE; and Group 4, high‐risk women who developed preterm PE.

### Maternal hemodynamic assessment

Maternal hemodynamic variables were measured and recorded at 12 + 0 to 15 + 6 weeks (Visit 1), 20 + 0 to 24 + 6 weeks (Visit 2) and 30 + 0 to 37 + 6 weeks' gestation (Visit 3). Blood pressure (BP) was measured simultaneously in both arms at 1‐min intervals for a total of four recordings, using validated automated devices (BP 3AQ1; Microlife, Taipei, Taiwan). MAP was calculated using the following formula: MAP = diastolic BP + (systolic BP − diastolic BP)/3^38^.

Heart rate (HR), stroke volume (SV), CO and systemic vascular resistance (SVR) were assessed using two‐dimensional (2D) transthoracic ultrasound (TTE). Participants were instructed to avoid caffeine, tea and alcoholic beverages for 24 h before the cardiac scan. 2D‐TTE was performed using either a Voluson E6 (GE Healthcare, Zipf, Austria) ultrasound machine, equipped with a phased‐array transducer (3SP‐D), or a V8 (Samsung Medison, Seoul, South Korea) ultrasound machine, equipped with a phased‐array transducer (PA1‐5A), according to the guidelines of the American Society of Echocardiography^39^. All echocardiographic scans were performed by research clinicians, who had been trained in image acquisition and analysis by a cardiologist (A.P.W.L.) with expertise in echocardiography. Our previous study reported good inter‐ and intraobserver reproducibility for hemodynamic measurements^16^. The transducer was positioned in the fourth intercostal space, just to the left of the sternum, to visualize the parasternal long‐axis view of the heart. The left ventricular outflow tract diameter (LVOT‐D) was measured 3–10 mm from the aortic annulus using the inner edge‐to‐inner edge method during midsystole. Two measurements were obtained and the mean LVOT‐D was recorded. Subsequently, the cross‐sectional area of the LVOT was calculated. Following this, the transducer was positioned at the cardiac apex and tilted toward the chest to visualize the five‐chamber view of the heart. Pulsed‐wave Doppler was used, with the selected sample volume placed about 5 mm proximal to the aortic valve. The angle of insonation was maintained at less than 20°. After obtaining three consecutive waveforms, the LVOT velocity–time integral (VTI) was measured for each waveform and the mean LVOT‐VTI was recorded. SV, CO and SVR were calculated based on the aforementioned measurements. SV was calculated as SV = π × (LVOT‐D/2)^2^ × LVOT‐VTI. CO was calculated as CO = SV × HR. SVR was calculated as SVR = MAP/CO × 80. The results of maternal hemodynamic assessments were not disclosed to the participants or their doctors and did not influence subsequent management of the pregnancy.

### Sample size calculation

The sample size was calculated using Glimmpse software (version 3.0.0; https://glimmpse.samplesizeshop.org/) for longitudinal studies, based on the changes in MAP during pregnancy among the four study groups. The expected means and SDs for MAP were derived from the original dataset of our previous study^40^, which employed the same measurement protocol and a similar study population. Assuming 80% statistical power, a two‐sided Type‐I error rate of 0.05 and a ratio of group sizes of 10 (Group 1):10 (Group 2):1 (Group 3):1 (Group 4), the minimum required sample sizes were estimated to be 220 for Group 1, 220 for Group 2, 22 for Group 3 and 22 for Group 4. Considering a 20% loss‐to‐follow‐up rate, approximately 275 low‐risk participants (Group 1) and 330 high‐risk participants (Groups 2, 3 and 4) were required for this study.

### Statistical analysis

Maternal demographic characteristics, medical history, obstetric history and pregnancy outcomes were compared between the four study groups. The normality of continuous variables was assessed using the Kolmogorov–Smirnov test. Non‐normally distributed continuous variables are presented as median (interquartile range). Categorical variables are presented as *n* (%). Non‐normally distributed continuous variables were compared using the Kruskal–Wallis test, followed by *post‐hoc* analysis. Categorical variables were compared using the chi‐square test or Fisher's exact test, as appropriate. Spearman's rank correlation was used to assess associations between maternal hemodynamic variables at each visit and neonatal outcomes.

As the distributions of maternal hemodynamic variables were non‐normal, HR, SV, CO, MAP and SVR were log_10_ transformed to approximate normality. Linear mixed‐effects analysis, including fixed and random effects, was performed for the repeated‐measures analysis of maternal hemodynamic variables, controlling for clinical visit (Visit 1, 2 and 3), study group (Group 1, 2, 3 and 4), maternal age, maternal weight, maternal height, previous pregnancy with PE, family history of PE, CH, pre‐existing diabetes mellitus Type 1 or 2, systemic lupus erythematosus (SLE) or antiphospholipid syndrome (APS), mode of conception, cigarette smoking status, and the interaction between study group and clinical visit. Linear mixed‐effects models were estimated using full information maximum likelihood, under the assumption of missing at random (MAR). Random‐effect components, including the intercept (participant identity) and slope (clinical visit), were considered in the models. The Akaike information criterion was used to define the best‐fit model (random slope only, random intercept only, or both random intercept and random slope) and to compare each model with the base model without random effects (Appendix S1)^41^. The best‐fit model was considered the primary model for all subsequent analyses. To evaluate the effect of CH, all participants with CH were excluded, and the primary model was refitted using only participants without CH. To assess the robustness of our primary findings to missing‐data mechanisms, we performed pattern‐mixture model analysis by incorporating the number of visits attended by each participant and its interactions with study group and clinical visit into the primary model.

*Post‐hoc* calculations of minimal detectable differences and effect sizes (Cohen's *d*) were performed for groupwise comparisons to evaluate power and precision. Multiple comparisons were adjusted using the Holm–Bonferroni correction and *P*‐values < 0.05 were considered statistically significant^42^. For interpretability, antilog‐transformed estimated marginal means, with absolute and percentage differences between study groups, are presented for each hemodynamic variable at each clinical visit.

Statistical analysis was performed using SPSS (version 29.0.1.0; IBM Corp., Armonk, NY, USA) and R statistical software (version 4.4.1 (2024); R Foundation for Statistical Computing, Vienna, Austria). Linear mixed‐effects models were fitted using the lmerTest package (version 3.1‐3) and lme4 package (version 1.1‐37).

## RESULTS

### Study population

**Figure 1:**
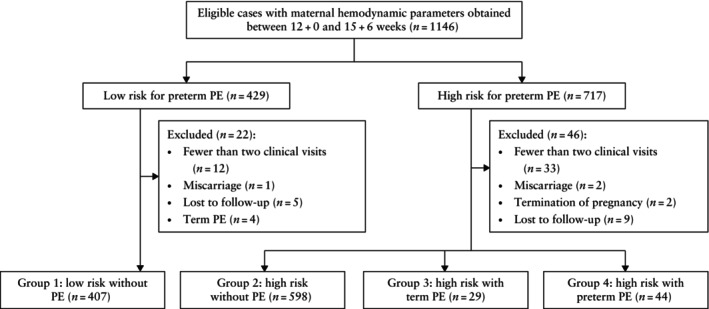
Flowchart summarizing inclusion of patients in study population. PE, pre‐eclampsia.

During the study period, a total of 717 high‐risk individuals and 429 matched low‐risk individuals agreed to participate in this longitudinal study (Figure 1). A total of 68 participants were excluded due to miscarriage (*n* = 3), termination of pregnancy (*n* = 2), loss to follow‐up (*n* = 14), fewer than two clinical visits (*n* = 45), or development of PE among low‐risk individuals (*n* = 4). The final analysis comprised 1078 participants, including 407 low‐risk women who did not develop PE (Group 1), 598 high‐risk women who did not develop PE (Group 2), 29 high‐risk women who developed term PE (Group 3) and 44 high‐risk women who developed preterm PE (Group 4). Baseline maternal characteristics were comparable between included and excluded cases (Table S1). There was no significant difference in the adjusted risk of preterm PE between included and excluded cases. Maternal clinical characteristics and pregnancy outcomes for the four study groups are presented in Table 1.

**Table 1:** Maternal characteristics and pregnancy outcomes of study population of Chinese women with singleton pregnancy (*n* = 1078), according to risk status for preterm pre‐eclampsia (PE) and subsequent development of PE

Variable	Group 1: low risk without PE (*n* = 407)	Group 2: high risk without PE (*n* = 598)	Group 3: high risk with term PE (*n* = 29)	Group 4: high risk with preterm PE (*n* = 44)	*P* *
Maternal age (years)	33.64 (31.22–36.16)^d^	34.34 (31.58–37.74)	34.08 (30.18–35.99)	35.10 (32.64–39.75)^a^	0.004
Height (cm)	160 (156–163)^b^	158 (155–162)^a^	160 (155–164)	158 (155–164)	< 0.001
Weight (kg)	55.7 (51.4–61.0)^b,c,d^	58.1 (51.8–65.8)^a^	57.4 (55.4–71.2)^a^	61.7 (56.2–70.6)^a^	< 0.001
Smoker at conception	16 (3.9)^b^	55 (9.2)^a,d^	1 (3.4)	0 (0)^b^	0.002
Mode of conception					0.427
Spontaneous	373 (91.6)	525 (87.8)	28 (96.6)	38 (86.4)	
*In‐vitro* fertilization	28 (6.9)	62 (10.4)	1 (3.4)	5 (11.4)	
Ovulation induction	6 (1.5)	11 (1.8)	0 (0)	1 (2.3)	
Parity	^b,c,d^	^a,d^	^a^	^a,b^	< 0.001
Nulliparous	256 (62.9)	440 (73.6)	21 (72.4)	24 (54.5)	
Parous with history of PE	147 (36.1)	125 (20.9)	5 (17.2)	6 (13.6)	
Parous without history of PE	4 (1.0)	33 (5.5)	3 (10.3)	14 (31.8)	
Chronic hypertension	0 (0)^b,c,d^	21 (3.5)^a,d^	3 (10.3)^a^	8 (18.2)^a,b^	< 0.001
Diabetes mellitus	1 (0.2)^d^	9 (1.5)^d^	1 (3.4)	5 (11.4)^a,b^	< 0.001
SLE/APS	0 (0)	7 (1.2)	1 (3.4)	1 (2.3)	0.052
Family history of PE	0 (0)^b,d^	13 (2.2)^a^	0 (0)	2 (4.5)^a^	0.007
GA at screening (days)	87 (85–89)	87 (85–89)	86 (84–88)	87 (85–89)	0.614
CRL at screening (mm)	59.2 (55.5–63.2)	59.1 (55.2–63.7)	58.0 (54.1–61.0)	59.1 (55.7–64.2)	0.595
MAP MoM	1.004 (0.957–1.060)^b,c,d^	1.101 (1.036–1.177)^a^	1.129 (1.035–1.218)^a^	1.124 (1.053–1.211)^a^	< 0.001
UtA‐PI MoM	0.984 (0.820–1.130)^b,c,d^	1.191 (1.021–1.367)^a^	1.119 (0.914–1.266)^a^	1.239 (1.009–1.444)^a^	< 0.001
PlGF MoM	1.079 (0.825–1.344)^b,c,d^	0.574 (0.400–0.779)^a^	0.520 (0.406–0.670)^a^	0.589 (0.374–0.774)^a^	< 0.001
Background PE risk (1: n)	234 (171–492)^b,c,d^	160 (102–232)^a,d^	154 (111–226)^a^	74 (27.5–151.5)^a,b^	< 0.001
Adjusted PE risk (1: n)	710 (298–1931)^b,c,d^	47 (25–71)^a,d^	29 (19–52)^a^	12 (5–26)^a,b^	< 0.001
Aspirin prophylaxis	0 (0)^b,c,d^	573 (95.8)^a^	29 (100)^a^	41 (93.2)^a^	< 0.001
Aspirin dose†					0.885
160 mg	—	571/573 (99.7)	29 (100)	41/41 (100)	
100 mg	—	2/573 (0.3)	0 (0)	0/41 (0)	
GA at aspirin initiation (weeks)†	—	13.7 (13.3–14.3)	13.7 (13.4–14.0)	14.0 (13.4–14.6)	0.096
Aspirin compliance (%)†	—	99.4 (96.8–100)	100 (96.3–100)	99.0 (94.5–100)	0.380
Mode of delivery	^c,d^	^d^	^a,d^	^a,b,c^	< 0.001
Non‐operative vaginal	214 (52.6)	273 (45.7)	10 (34.5)	6 (13.6)	
Operative vaginal (forceps/vacuum)	24 (5.9)	39 (6.5)	5 (17.2)	0 (0)	
Cesarean section	169 (41.5)	286 (47.8)	14 (48.3)	38 (86.4)	
Stillbirth or neonatal death	0 (0)	3 (0.5)	0 (0)	1 (2.3)	0.102
Composite neonatal morbidity‡	16 (3.9)^d^	27 (4.5)^d^	1 (3.4)	6 (13.6)^a,b^	0.035
GA at delivery (days)	272 (267–278)^b,c,d^	269 (265–275)^a,d^	266 (260–269)^a,d^	244 (232–253)^a,b,c^	< 0.001
Birth weight (g)	3105 (2858–3365)^b,d^	2920 (2670–3163)^a^	2785 (2490–3065)	1870.5 (1575–2305)^a^	< 0.001
Birth‐weight percentile	46.1 (23.4–72.3)^b,c,d^	31.2 (11.5–60.9)^a,d^	29.6 (6.3–45.9)^a,d^	2.0 (0.2–10.1)^a,b,c^	< 0.001
1‐min Apgar score < 7	9/369 (2.4)^b,d^	32/546 (5.9)^a,d^	2/28 (7.1)^d^	16 (36.4)^a,b,c^	< 0.001
5‐min Apgar score < 7	0/369 (0)^d^	5/546 (0.9)^d^	0/28 (0)	5 (11.4)^a,b^	< 0.001
Cord arterial pH§	7.27 (7.22–7.30)	7.26 (7.21–7.30)	7.27 (7.16–7.32)	7.28 (7.24–7.31)	0.467
Cord arterial base excess (mmol/L)¶	−5.0 (−7.0 to −3.0)	−5.0 (−7.0 to −3.0)^d^	−5.0 (−9.0 to −3.0)	−3.5 (−6.0 to −2.0)^b^	0.009
Cord venous pH**	7.33 (7.28–7.36)	7.31 (7.28–7.35)	7.31 (7.22–7.34)	7.31 (7.28–7.34)	0.016
Male sex	212 (52.1)	333 (55.7)	13 (44.8)	24 (54.5)	0.518

Data are given as median (interquartile range), *n* (%) or *n/N* (%).

* Overall *P*‐value for four‐way comparison; statistically significant (*P* < 0.05) pairwise comparisons are indicated by superscript letters, where ^a^ is comparison with Group 1, ^b^ is comparison with Group 2, ^c^ is comparison with Group 3 and ^d^ is comparison with Group 4.

† Data pertain only to patients who received aspirin prophylaxis.

‡ Composite neonatal morbidity included intraventricular hemorrhage ≥ Grade II, neonatal sepsis confirmed by culture, anemia resulting in blood transfusion, respiratory distress syndrome requiring surfactant and ventilation, and necrotizing enterocolitis requiring surgical intervention.

§ Data available for 747 participants.

¶ Data available for 764 participants.

** Data available for 743 participants. APS, antiphospholipid syndrome; CRL, crown–rump length; GA, gestational age; MAP, mean arterial pressure; MoM, multiples of the median; UtA‐PI, uterine artery pulsatility index; PlGF, placental growth factor; SLE, systemic lupus erythematosus.

### Multilevel linear mixed‐effects models

**Figure 2:**
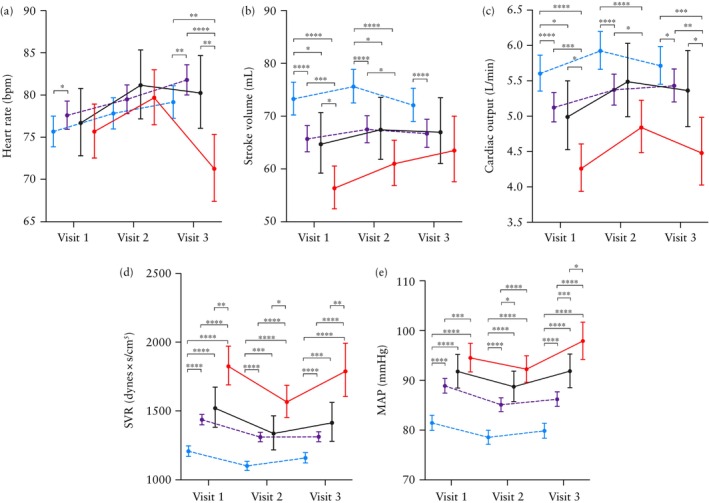
Temporal changes in estimated marginal means (antilog values) for maternal hemodynamic variables throughout gestation: (a) heart rate; (b) stroke volume; (c) cardiac output; (d) systemic vascular resistance (SVR); and (e) mean arterial pressure (MAP). Error bars are 95% CI. Statistically significant results are shown for pairwise comparisons between groups at each visit: 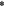 *P* < 0.05, 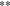 *P* < 0.01, 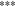 *P* < 0.001, 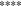 *P* < 0.0001. 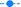, Low risk without pre‐eclampsia (PE) (Group 1); 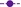, high risk without PE (Group 2); 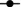, high risk with term PE (Group 3); 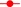, high risk with preterm PE (Group 4). Visit 1, 12 + 0 to 15 + 6 weeks; Visit 2, 20 + 0 to 24 + 6 weeks; Visit 3, 30 + 0 to 37 + 6 weeks.

The fixed effects of the best multilevel models are shown in Tables S2 and S3. For all maternal hemodynamic variables, a random intercept–random slope model provided the best fit to the data (Appendix S1). The estimated marginal means (antilog transformed), with 95% CI, and the corresponding absolute and percentage differences between study groups for all maternal hemodynamic variables are presented in Tables 2, 3 and S4, as well as in Figure 2. The estimated marginal mean differences (with 95% CI), Cohen's *d* effect sizes (with 95% CI) and minimal detectable differences are presented in Table S5. Aspirin prophylaxis was not included as a covariate, as it was prescribed routinely to high‐risk women, with no significant differences in use, dosage or compliance across high‐risk groups (Table 1). Furthermore, research has shown a limited effect of aspirin prophylaxis on maternal hemodynamics during pregnancy^43^.

**Table 2:** Estimated marginal means (antilog values) for maternal hemodynamic variables, derived from multilevel linear mixed‐effects models, across clinical visits and study groups

Hemodynamic variable	Group 1: low risk without PE (*n* = 407)	Group 2: high risk without PE (*n* = 598)	Group 3: high risk with term PE (*n* = 29)	Group 4: high risk with preterm PE (*n* = 44)	*P* (between groups)	*P* (throughout pregnancy)
Heart rate						0.0002
Visit 1 (bpm)	75.68 (73.89–77.51)^b^	77.60 (75.95–79.29)^a^	76.70 (72.82–80.78)	75.67 (72.55–78.92)	0.0332	
Visit 2 (bpm)	77.81 (75.99–79.68)	79.48 (77.81–81.20)	81.15 (77.17–85.33)	79.67 (76.49–82.99)	0.0495	
Visit 3 (bpm)	79.15 (77.25–81.09)^b,d^	81.78 (80.02–83.57)^a,d^	80.25 (76.08–84.65)^d^	71.27 (67.43–75.34)^a,b,c^	< 0.0001	
*P* (between visits)	< 0.0001	< 0.0001	0.0583	0.0001		
Visit 1 *vs* Visit 2	< 0.0001	< 0.0001	NS	< 0.05		
Visit 1 *vs* Visit 3	< 0.0001	< 0.0001	NS	< 0.05		
Visit 2 *vs* Visit 3	< 0.05	< 0.0001	NS	0.0001		
Stroke volume						< 0.0001
Visit 1 (mL)	73.29 (70.26–76.44)^b,c,d^	65.71 (63.27–68.25)^a,d^	64.72 (59.24–70.72)^a,d^	56.38 (52.48–60.57)^a,b,c^	< 0.0001	
Visit 2 (mL)	75.63 (72.51–78.88)^b,c,d^	67.50 (64.98–70.13)^a,d^	67.46 (61.84–73.60)^a^	61.03 (56.87–65.49)^a,b^	< 0.0001	
Visit 3 (mL)	72.10 (69.01–75.32)^b^	66.72 (64.10–69.44)^a^	67.00 (61.06–73.52)	63.50 (57.60–70.01)	< 0.0001	
*P* (between visits)	0.0004	0.0263	0.6249	0.0251		
Visit 1 *vs* Visit 2	< 0.05	< 0.05	NS	NS		
Visit 1 *vs* Visit 3	NS	NS	NS	NS		
Visit 2 *vs* Visit 3	< 0.001	NS	NS	NS		
Cardiac output						< 0.0001
Visit 1 (L/min)	5.602 (5.354–5.861)^b,c,d^	5.121 (4.918–5.334)^a,d^	4.989 (4.526–5.498)^a,d^	4.258 (3.936–4.607)^a,b,c^	< 0.0001	
Visit 2 (L/min)	5.922 (5.662–6.195)^b,d^	5.371 (5.157–5.594)^a,d^	5.486 (4.992–6.029)	4.840 (4.484–5.225)^a,b^	< 0.0001	
Visit 3 (L/min)	5.710 (5.450–5.983)^b,d^	5.429 (5.202–5.665)^a,d^	5.362 (4.851–5.927)^d^	4.479 (4.026–4.984)^a,b,c^	< 0.0001	
*P* (between visits)	0.0002	< 0.0001	0.1586	0.0071		
Visit 1 *vs* Visit 2	0.0001	0.0001	NS	< 0.01		
Visit 1 *vs* Visit 3	NS	< 0.0001	NS	NS		
Visit 2 *vs* Visit 3	< 0.05	NS	NS	NS		
SVR						< 0.0001
Visit 1 (dynes × s/cm^5^)	1208.2 (1171.1–1246.5)^b,c,d^	1437.2 (1399.4–1476.0)^a,d^	1520.7 (1382.3–1672.9)^a,d^	1824.7 (1689.8–1970.3)^a,b,c^	< 0.0001	
Visit 2 (dynes × s/cm^5^)	1101.3 (1068.0–1135.6)^b,c,d^	1311.1 (1277.1–1345.9)^a,d^	1336.3 (1218.2–1465.8)^a,d^	1565.8 (1453.6–1686.8)^a,b,c^	< 0.0001	
Visit 3 (dynes × s/cm^5^)	1160.0 (1123.1–1198.0)^b,c,d^	1312.5 (1277.1–1348.9)^a,d^	1414.8 (1281.0–1562.6)^a,d^	1788.5 (1606.2–1991.6)^a,b,c^	< 0.0001	
*P* (between visits)	< 0.001	< 0.001	0.046	< 0.001		
Visit 1 *vs* Visit 2	< 0.0001	< 0.0001	< 0.05	< 0.001		
Visit 1 *vs* Visit 3	< 0.01	< 0.0001	NS	NS		
Visit 2 *vs* Visit 3	< 0.001	NS	NS	< 0.05		
MAP						< 0.0001
Visit 1 (mmHg)	81.52 (80.03–83.04)^b,c,d^	88.99 (87.53–90.47)^a,d^	91.88 (88.55–95.33)^a^	94.63 (91.78–97.56)^a,b^	< 0.0001	
Visit 2 (mmHg)	78.62 (77.20–80.06)^b,c,d^	85.19 (83.81–86.59)^a,c,d^	88.83 (85.81–91.96)^a,b^	92.34 (89.72–95.04)^a,b^	< 0.0001	
Visit 3 (mmHg)	79.93 (78.45–81.44)^b,c,d^	86.30 (84.85–87.78)^a,c,d^	91.95 (88.63–95.41)^a,b,d^	98.01 (94.33–101.83)^a,b,c^	< 0.0001	
*P* (between visits)	< 0.0001	< 0.0001	0.0344	0.0020		
Visit 1 *vs* Visit 2	< 0.0001	< 0.0001	NS	NS		
Visit 1 *vs* Visit 3	< 0.001	< 0.0001	NS	NS		
Visit 2 *vs* Visit 3	< 0.001	< 0.001	NS	< 0.01		

Data in parentheses are 95% CI. Statistically significant (*P* < 0.05) pairwise comparisons between groups are indicated by superscript letters, where ^a^ is comparison with Group 1, ^b^ is comparison with Group 2, ^c^ is comparison with Group 3 and ^d^ is comparison with Group 4. Significance levels for group comparisons are shown in Figure 2 and Table S4. MAP, mean arterial pressure; NS, not significant; PE, pre‐eclampsia; SVR, systemic vascular resistance; Visit 1, 12 + 0 to 15 + 6 weeks; Visit 2, 20 + 0 to 24 + 6 weeks; Visit 3, 30 + 0 to 37 + 6 weeks.

**Table 3:** Absolute and percentage differences in estimated marginal means (antilog values) for maternal hemodynamic variables, across clinical visits and study groups

	Heart rate		Stroke volume		Cardiac output		SVR		MAP	
Visit/group	Absolute difference (bpm)	Percentage difference (%)	Absolute difference (mL)	Percentage difference (%)	Absolute difference (L/min)	Percentage difference (%)	Absolute difference (dynes × s/cm^5^)	Percentage difference (%)	Absolute difference (mmHg)	Percentage difference (%)
Visit 1										
Group 1 *vs* Group 2	1.9 (−0.5 to 4.4)	2.5 (0.2 to 4.9)	−7.6 (−11.5 to −3.6)	−10.3 (−13.9 to −6.7)	−0.48 (−0.81 to −0.15)	−8.6 (−12.5 to −4.5)	229.0 (175.4 to 282.7)	19.0 (13.8 to 24.4)	7.5 (5.4 to 9.6)	9.2 (7.4 to 11.0)
Group 1 *vs* Group 3	1.0 (−3.3 to 5.4)	1.3 (−5.4 to 8.6)	−8.6 (−15.1 to −2.1)	−11.7 (−21.5 to −0.7)	−0.61 (−1.16 to −0.07)	−10.9 (−21.8 to −1.4)	312.5 (162.7 to 462.3)	25.9 (10.3 to 43.6)	10.4 (6.7 to 14.1)	12.7 (7.4 to 18.2)
Group 1 *vs* Group 4	0.0 (−3.7 to 3.6)	0.0 (−5.6 to 5.9)	−16.9 (−22.0 to −11.8)	−23.1 (−30.4 to −14.9)	−1.34 (−1.76 to −0.92)	−24.0 (−31.9 to −15.1)	616.5 (471.5 to 761.5)	51.0 (35.1 to 68.8)	13.1 (9.4 to 16.8)	16.1 (11.5 to 20.8)
Group 2 *vs* Group 3	−0.9 (−5.2 to 3.4)	−1.2 (−7.7 to 5.8)	−1.0 (−7.2 to 5.3)	−1.5 (−12.3 to 10.6)	−0.13 (−0.66 to 0.40)	−2.6 (−14.3 to 10.7)	83.4 (−66.5 to 233.4)	5.8 (−7.1 to 20.5)	2.9 (−0.8 to 6.6)	3.2 (−1.5 to 8.2)
Group 2 *vs* Group 4	−1.9 (−5.5 to 1.7)	−2.5 (−7.8 to 3.2)	−9.3 (−14.1 to −4.6)	−14.2 (−22.2 to −5.4)	−0.86 (−1.26 to −0.47)	−16.8 (−25.3 to −7.4)	387.5 (242.4 to 532.6)	27.0 (13.9 to 41.6)	5.6 (2.4 to 8.9)	6.3 (2.2 to 10.6)
Group 3 *vs* Group 4	−1.0 (−6.1 to 4.1)	−1.3 (−9.5 to 7.5)	−8.3 (−15.4 to −1.3)	−12.9 (−24.7 to 0.8)	−0.73 (−1.32 to −0.14)	−14.6 (−27.4 to −0.3)	304.0 (102.5 to 505.6)	20.0 (1.8 to 41.5)	2.8 (−1.7 to 7.2)	3.0 (−2.9 to 9.3)
Visit 2										
Group 1 *vs* Group 2	1.7 (−0.8 to 4.2)	2.1 (−0.1 to 4.5)	−8.1 (−12.2 to −4.0)	−10.7 (−14.2 to −7.2)	−0.55 (−0.90 to −0.21)	−9.3 (−13.1 to −5.3)	209.7 (161.6 to 257.9)	19.0 (14.0 to 24.3)	6.6 (4.6 to 8.6)	8.4 (6.7 to 10.0)
Group 1 *vs* Group 3	3.3 (−1.1 to 7.8)	4.3 (−1.5 to 11.5)	−8.2 (−14.8 to −1.5)	−10.8 (−20.5 to 0.1)	−0.44 (−1.02 to 0.15)	−7.4 (−18.3 to 5.0)	235.0 (106.9 to 363.0)	21.3 (6.8 to 37.9)	10.2 (6.8 to 13.6)	13.0 (8.1 to 18.1)
Group 1 *vs* Group 4	1.9 (−1.9 to 5.6)	2.4 (−3.2 to 8.3)	−14.6 (−20.0 to −9.3)	−19.3 (−26.9 to −11.0)	−1.08 (−1.54 to −0.63)	−18.3 (−26.5 to −9.1)	464.5 (343.3 to 585.8)	42.2 (27.6 to 58.4)	13.7 (10.7 to 16.7)	17.5 (13.2 to 21.9)
Group 2 *vs* Group 3	1.7 (−2.7 to 6.1)	2.1 (−4.4 to 9.0)	0.0 (−6.5 to 6.4)	−0.1 (−10.8 to 11.9)	0.12 (−0.45 to 0.68)	2.1 (−9.7 to 15.5)	25.2 (−103.0 to 153.4)	1.9 (−10.2 to 15.6)	3.6 (0.3 to 7.0)	4.3 (−0.2 to 8.9)
Group 2 *vs* Group 4	0.2 (−3.5 to 3.8)	0.2 (−5.1 to 5.9)	−6.5 (−11.5 to −1.5)	−9.6 (−17.8 to −0.5)	−0.53 (−0.96 to −0.10)	−9.9 (−18.8 to −0.1)	254.8 (133.4 to 376.2)	19.4 (7.5 to 32.7)	7.2 (4.2 to 10.2)	8.4 (4.5 to 12.4)
Group 3 *vs* Group 4	−1.5 (−6.7 to 3.7)	−1.8 (−9.6 to 6.7)	−6.4 (−13.7 to 0.8)	−9.5 (−21.6 to 4.4)	−0.65 (−1.28 to −0.01)	−11.8 (−24.5 to 3.1)	229.6 (59.8 to 399.3)	17.2 (−0.1 to 37.4)	3.5 (−0.5 to 7.6)	4.0 (−1.6 to 9.8)
Visit 3										
Group 1 *vs* Group 2	2.6 (0.0 to 5.2)	3.3 (0.9 to 5.8)	−5.4 (−9.5 to −1.3)	−7.5 (−11.3 to −3.5)	−0.28 (−0.63 to 0.07)	−4.9 (−9.2 to 0.5)	152.6 (100.7 to 204.4)	13.2 (8.0 to 18.6)	6.4 (4.3 to 8.5)	8.0 (6.2 to 9.7)
Group 1 *vs* Group 3	1.1 (−3.6 to 5.8)	1.4 (−5.6 to 8.9)	−5.1 (−12.1 to 1.9)	−7.1 (−17.8 to 5.1)	−0.35 (−0.95 to 0.25)	−6.1 (−17.8 to 7.2)	254.9 (109.6 to 400.2)	22.0 (6.3 to 40.0)	12.0 (8.3 to 15.7)	15.0 (9.7 to 20.6)
Group 1 *vs* Group 4	−7.9 (−12.3 to −3.5)	−9.9 (−16.5 to −2.9)	−8.6 (−15.5 to −1.7)	−11.9 (−23.0 to 0.7)	−1.23 (−1.78 to −0.68)	−21.6 (−32.2 to −9.2)	628.6 (432.8 to 824.4)	54.2 (32.5 to 79.5)	18.1 (14.0 to 22.1)	22.6 (16.6 to 28.9)
Group 2 *vs* Group 3	−1.5 (−6.2 to 3.1)	−1.9 (−8.5 to 5.3)	0.3 (−6.5 to 7.0)	0.4 (−11.0 to 13.3)	−0.07 (−0.65 to 0.52)	−1.2 (−13.3 to 12.6)	102.3 (−42.6 to 247.2)	7.8 (−5.9 to 23.5)	5.7 (2.0 to 9.3)	6.6 (1.7 to 11.6)
Group 2 *vs* Group 4	−10.5 (−14.8 to −6.2)	−12.8 (−19.1 to −6.1)	−3.2 (−10.0 to 3.5)	−4.8 (−16.5 to 8.6)	−0.95 (−1.48 to −0.42)	−17.5 (−28.6 to −4.7)	476.0 (280.5 to 671.6)	36.3 (17.3 to 58.3)	11.7 (7.7 to 15.7)	13.6 (8.1 to 19.3)
Group 3 *vs* Group 4	−9.0 (−14.8 to −3.2)	−11.2 (−19.6 to −1.8)	−3.5 (−12.3 to 5.3)	−5.2 (−20.4 to 12.8)	−0.88 (−1.6 to −0.16)	−16.5 (−30.9 to 1.0)	373.7 (135.7 to 611.7)	26.4 (3.8 to 54.0)	6.1 (1.0 to 11.1)	6.6 (−0.2 to 13.9)

Data in parentheses are 95% CI. Group 1, low risk without pre‐eclampsia (PE); Group 2, high risk without PE; Group 3, high risk with term PE; Group 4, high risk with preterm PE; MAP, mean arterial pressure; SVR, systemic vascular resistance; Visit 1, 12 + 0 to 15 + 6 weeks; Visit 2, 20 + 0 to 24 + 6 weeks; Visit 3, 30 + 0 to 37 + 6 weeks.

Increasing maternal height was associated with higher log_10_ SV and log_10_ CO, and lower log_10_ MAP and log_10_ SVR (Tables S2 and S3). Increasing maternal weight was associated with higher log_10_ SV, log_10_ CO and log_10_ MAP. CH was associated with higher log_10_ HR, log_10_ SV, log_10_ CO and log_10_ MAP. Smoking at conception was associated with lower log_10_ MAP. Compared with nulliparous women, parous women without a history of PE had higher log_10_ SV and log_10_ CO and lower log_10_ SVR, while those with a history of PE had higher log_10_ CO. Compared with Group 1, Group 2 was associated with higher log_10_ HR, and Groups 2–4 were associated with lower log_10_ SV and log_10_ CO, but higher log_10_ SVR and log_10_ MAP. Clinical visit was associated with all hemodynamic variables. Specifically, compared with Visit 1, both Visit 2 and Visit 3 were associated with higher log_10_ HR and lower log_10_ SVR and log_10_ MAP, while only Visit 2 was associated with higher log_10_ SV and log_10_ CO. Analysis of group–visit interactions indicated that, at Visit 3, Group 4 had lower log_10_ HR and higher log_10_ SV and log_10_ MAP compared with Group 1, whereas Group 2 exhibited a higher log_10_ CO and lower log_10_ SVR compared with Group 1. There was no significant contribution to any of the maternal hemodynamic variables from maternal age, family history of PE, diabetes mellitus Type 1 or 2, SLE/APS or mode of conception. Excluding women with CH did not materially alter the size of the fixed effects of maternal characteristics and study groups (Table S6).

The number of participants included in the hemodynamic analysis at each clinical visit is summarized in Table S7. Sensitivity analysis using a pattern‐mixture model showed no significant interactions between study group and the number of visits attended for most groups and hemodynamic variables, with the exception of an effect on log_10_ HR in high‐risk women with term PE (β = 0.062; *P* = 0.049) (Table S8). However, this group had a limited sample size (only 3/29 participants did not undergo all three scans), limiting interpretation.

### Temporal changes in estimated marginal means of hemodynamic variables throughout gestation

#### Heart rate

HR exhibited a significant upward trend with advancing gestation in Groups 1 and 2 (Tables 2 and S4, Figure 2a). Notably, Group 2 demonstrated a significantly higher HR throughout gestation compared with Group 1. Although both Groups 3 and 4 showed an increasing trend in HR from Visit 1 to Visit 2, a subsequent decline was observed from Visit 2 to Visit 3. The decline was most pronounced in Group 4, resulting in the lowest HR among the four groups at 30 + 0 to 37 + 6 weeks. Compared with Group 1, HR in Group 4 differed by −7.9 (95% CI, −12.3 to −3.5) bpm at Visit 3, corresponding to a percentage difference of −9.9% (95% CI, −16.5% to −2.9%) (Table 3).

#### Stroke volume

In Group 1, SV showed a slight increase from Visit 1 to Visit 2, followed by a subsequent decrease from Visit 2 to Visit 3 (Tables 2 and S4, Figure 2b). Compared with Group 1, Groups 2 and 3 maintained a significantly lower SV throughout gestation, with no significant difference observed between the latter two groups. Group 4 consistently showed the lowest SV throughout gestation, with marked and significant differences already apparent at Visit 1 compared with the other three groups. Compared with Group 1, SV in Group 4 differed by −16.9 (95% CI, −22.0 to −11.8) mL at Visit 1, corresponding to a percentage difference of −23.1% (95% CI, −30.4% to −14.9%) (Table 3). These differences gradually diminished with advancing gestation and were no longer statistically significant at 30 + 0 to 37 + 6 weeks.

#### Cardiac output

In Groups 1, 3 and 4, CO increased to its peak at Visit 2 (Tables 2 and S4, Figure 2c). In contrast, Group 2 exhibited a consistent increase in CO throughout gestation. Compared with Group 1, all high‐risk groups showed a significantly lower CO throughout gestation, with Group 4 exhibiting the lowest values at all visits. Specifically, compared with Group 1, CO in Group 4 differed by −1.34 (95% CI, −1.76 to −0.92) L/min at Visit 1, −1.08 (95% CI, −1.54 to −0.63) L/min at Visit 2 and −1.23 (95% CI, −1.78 to −0.68) L/min at Visit 3, corresponding to percentage differences of −24.0% (95% CI, −31.9% to −15.1%), −18.3% (95% CI, −26.5% to −9.1%) and −21.6% (95% CI, −32.2% to −9.2%), respectively (Table 3).

#### Systemic vascular resistance

In Groups 1, 3 and 4, SVR showed a decreasing trend from Visit 1 to Visit 2, followed by an increase from Visit 2 to Visit 3 (Tables 2 and S4, Figure 2d). SVR in Group 2 also decreased from Visit 1 to Visit 2, but then plateaued from Visit 2 to Visit 3. Group 1 exhibited a significantly lower SVR compared with Groups 2, 3 and 4 throughout pregnancy. Specifically, SVR in Group 4 was higher than that in Group 1 by 616.5 (95% CI, 471.5–761.5) dynes × s/cm^5^ at Visit 1, 464.5 (95% CI, 343.3–585.8) dynes × s/cm^5^ at Visit 2 and 628.6 (95% CI, 432.8–824.4) dynes × s/cm^5^ at Visit 3, corresponding to percentage differences of 51.0% (95% CI, 35.1–68.8%), 42.2% (95% CI, 27.6–58.4%) and 54.2% (95% CI, 32.5–79.5%), respectively (Table 3). There was no significant difference in SVR between Groups 2 and 3, while both groups had significantly lower SVR compared with Group 4.

#### Mean arterial pressure

MAP exhibited a V‐shaped trajectory, declining from Visit 1 to Visit 2, and then increasing toward Visit 3 in all study groups (Tables 2 and S4, Figure 2e). Throughout gestation, MAP consistently ranked from highest to lowest as follows: Group 4, Group 3, Group 2, Group 1. Specifically, MAP in Group 4 was higher than that in Group 1 by 13.1 (95% CI, 9.4–16.8) mmHg at Visit 1, 13.7 (95% CI, 10.7–16.7) mmHg at Visit 2 and 18.1 (95% CI, 14.0–22.1) mmHg at Visit 3, corresponding to percentage differences of 16.1% (95% CI, 11.5–20.8%), 17.5% (95% CI, 13.2–21.9%) and 22.6% (95% CI, 16.6–28.9%), respectively (Table 3). Moreover, the differences in MAP among the three high‐risk groups became increasingly pronounced with advancing gestational age.

### Correlation between hemodynamic profile and pregnancy outcome

The correlations of maternal hemodynamic variables with 1‐min and 5‐min Apgar scores, cord arterial pH, cord arterial base excess, cord venous pH and birth weight are shown in Table 4. Increased HR, SV and CO were correlated weakly with increased birth weight, whereas increased SVR and MAP were correlated weakly with decreased birth weight. Both SV and CO showed weak positive correlations with cord venous pH, while SVR exhibited a weak negative correlation. Elevated MAP was correlated weakly with lower 1‐min Apgar score throughout gestation and with lower 5‐min Apgar score at Visits 2 and 3.

**Table 4:** Correlation between log_10_ maternal hemodynamic variables and pregnancy outcomes in study population, according to clinical visit

Log_10_ hemodynamic variable	1‐min Apgar score	5‐min Apgar score	Cord arterial pH	Cord arterial base excess (in mmol/L)	Cord venous pH	Birth weight (in g)
Heart rate (in bpm)						
Visit 1	−0.027	−0.001	−0.078*	−0.030	−0.011	0.074*
Visit 2	−0.033	−0.001	−0.032	−0.044	−0.003	0.064*
Visit 3	0.018	0.011	−0.078*	−0.046	0.023	0.148**
Stroke volume (in mL)						
Visit 1	−0.015	0.018	0.028	0.045	0.121**	0.105**
Visit 2	−0.011	−0.012	0.029	0.060	0.107**	0.108**
Visit 3	−0.041	−0.057	0.064	0.089*	0.081*	0.130**
Cardiac output (in L/min)						
Visit 1	−0.021	0.018	−0.007	0.046	0.110**	0.132**
Visit 2	−0.019	−0.009	0.021	0.041	0.103**	0.129**
Visit 3	−0.032	−0.054	0.025	0.064	0.099*	0.188**
SVR (in dynes × s/cm^5^)						
Visit 1	−0.011	−0.037	−0.008	−0.065	−0.128**	−0.183**
Visit 2	−0.035	−0.031	−0.006	−0.040	−0.122**	−0.182**
Visit 3	−0.011	0.007	−0.018	−0.045	−0.110**	−0.238**
MAP (in mmHg)						
Visit 1	−0.102**	−0.056	−0.043	−0.072*	−0.070	−0.111**
Visit 2	−0.128**	−0.082*	−0.013	−0.032	−0.063	−0.122**
Visit 3	−0.111**	−0.091**	0.012	0.012	−0.046	−0.143**

Values are Spearman's rank correlation coefficient.

* *P* < 0.05.

** *P* < 0.01. MAP, mean arterial pressure; SVR, systemic vascular resistance; Visit 1, 12 + 0 to 15 + 6 weeks; Visit 2, 20 + 0 to 24 + 6 weeks; Visit 3, 30 + 0 to 37 + 6 weeks.

## DISCUSSION

### Main findings

This longitudinal study demonstrates that women identified as high‐risk for preterm PE by the first‐trimester FMF combined test exhibit distinct hemodynamic profiles depending on whether they subsequently develop preterm PE, term PE or do not develop PE. Key findings include, first, that high‐risk women without PE exhibit mildly decreased CO (characterized by slightly elevated HR and consistently lower SV) compared with low‐risk controls, alongside persistently higher SVR and MAP. Second, high‐risk women who develop term PE exhibit a similar hypodynamic profile to high‐risk women without PE, but with persistently elevated MAP from midgestation onwards. Third, high‐risk women who develop preterm PE exhibit a sustained reduction in CO from early gestation onwards, driven by the lowest SV in early to mid pregnancy and a late‐gestation decline in HR. SVR and MAP were consistently elevated in these women compared with the other groups and the differences became more pronounced as gestation advanced. Fourth, there were weak correlations between hemodynamic variables and pregnancy outcomes.

### Comparison with previous studies and interpretation of findings

CO, determined by the product of SV and HR, has been investigated increasingly in the context of maternal cardiac adaptation among women who develop PE during pregnancy. Easterling *et al*.^30^ described a persistently hyperdynamic disease model for PE, whereas Bosio *et al*.^27^ described a late crossover from hyperdynamic to hypodynamic circulation. Two other studies found no significant difference in CO between women with PE and controls^13^, ^26^. Valensise *et al*.^17^ suggested that early PE was associated with decreased CO, while late PE was associated with increased CO. However, these studies were limited by their small sample sizes and/or lack of adjustment for maternal characteristics. A larger longitudinal study of 1789 unselected pregnancies showed that, compared with normotensive women and after controlling for maternal characteristics, women with PE had higher CO, driven by greater SV in the first trimester, but transitioned to a state of low CO and high peripheral vascular resistance after 20 weeks' gestation^29^. A substudy of the Combined Multimarker Screening and Randomized Patient Treatment with Aspirin for Evidence‐Based Pre‐eclampsia Prevention (ASPRE) trial found no significant difference in CO between high‐risk women who developed PE and those who did not^43^. However, these two studies were limited by the lack of stratification by PE subtype^29^, ^43^. This study found that high‐risk women, irrespective of whether they subsequently developed preterm PE, term PE or did not develop PE, exhibited persistent and varying degrees of hypodynamic circulation throughout pregnancy compared with low‐risk controls. Our findings provide trimester‐ and subtype‐specific insights, suggesting that the markedly reduced CO in women with preterm PE results from a severely decreased SV in early to mid gestation and a substantial decline in HR in late gestation. By contrast, the mild reduction in CO in high‐risk women with term PE and those without PE was due to a mild decrease in SV in concert with a normal or slightly elevated HR, respectively.

The distinct hemodynamic profile in high‐risk women may reflect impaired regulatory mechanisms. Early SV reduction could be related to insufficient plasma volume expansion or compromised myocardial contractility, potentially linked to endothelial dysfunction, abnormal placentation or pre‐existing subclinical cardiac strain^14^, ^40^, ^44^, ^45^. The late‐gestation HR decline in women with preterm PE, compared to a slight increase in high‐risk women without PE and an intermediate state in those with term PE, suggests a possible alteration in autonomic regulation to maintain CO under increasing hemodynamic load^46^, ^47^. This impaired chronotropic response may be associated with both the development and severity of PE. Future studies incorporating direct physiological assessments, such as validated plasma volume estimation, echocardiography and formal autonomic testing (e.g. HR variability), are warranted to clarify which of hypovolemia, myocardial contractile impairment or autonomic dysfunction is the primary driver of these observed trajectories.

Previous authors have reported that SVR, another key determinant of MAP alongside CO, is elevated in cases of PE^16^, ^45^, ^48^. Our findings corroborate this pattern: SVR was highest in high‐risk women who developed preterm PE from early gestation onwards, followed by high‐risk women with term PE and then high‐risk women without PE. This suggests that elevated SVR in high‐risk women may be associated specifically with the development of preterm PE, rather than PE in general. Our group reported previously that MAP was highest in high‐risk women with PE, followed by high‐risk women without PE, and lowest in the low‐risk group^40^. In this study, we further demonstrated that MAP was consistently highest in the preterm PE group, followed by the term PE group and then the high‐risk group without PE, with differences becoming more pronounced as gestation advanced.

### Clinical implications

These findings support the value of early and serial hemodynamic monitoring for risk stratification. The findings of low SV in early gestation, declining HR in late gestation and persistently elevated SVR and MAP throughout pregnancy may serve as early warning signs for preterm PE. Distinct hemodynamic profiles in preterm and term PE reinforce the concept of phenotypic subtypes and may guide tailored surveillance and intervention. Moreover, persistent cardiovascular maladaptation in high‐risk women without PE throughout pregnancy indicates an increased risk of long‐term cardiovascular morbidity. This underscores the need for further research into the long‐term cardiovascular health of these women, including postpartum risk assessment.

### Strengths and limitations

The strengths of this study include: its prospective longitudinal design within the FMF first‐trimester combined‐test framework^7^, ^8^, ^9^, ^10^; stratification of high‐risk women by PE subtype; and the use of TTE, a widely accepted and reliable method for non‐invasive cardiac assessment^25^. Additionally, linear mixed‐effects models were used to adjust for variables that may influence the hemodynamic profile, such as maternal demographic characteristics and CH.

This study has several limitations that should be acknowledged. First, PE was identified only among high‐risk women, and this was a single‐center study conducted in a Chinese population, which may limit generalizability. Second, serial measurements of PlGF and UtA‐PI were not available, preventing mechanistic integration of placental dysfunction with maternal hemodynamics^19^. Future multicenter, multiethnic longitudinal studies with integrated placental–cardiac assessment in a non‐selected population are warranted to clarify the interplay between these mechanisms. Third, the sample size, calculated based on MAP, may not provide sufficient power for other variables; thus, between‐group comparisons, particularly those involving Group 3, should be considered exploratory. Nonetheless, effect sizes for the smallest groups (Groups 3 and 4) were generally moderate to large, indicating that meaningful differences could still be detected.

### Conclusions

In this study, we have demonstrated distinct hemodynamic profiles throughout pregnancy in high‐risk women who subsequently develop preterm PE, term PE or do not develop PE compared with low‐risk women who do not develop PE. These findings highlight a multifactorial mechanism underlying the development of PE subtypes at different stages of pregnancy and underscore the importance of longitudinal hemodynamic monitoring for early identification of PE, refined risk stratification and development of targeted preventive strategies.

## Supporting information


**Appendix S1** Mixed‐effects model selection.
**Table S1** Comparison of baseline maternal characteristics between included and excluded cases.
**Table S2** Fixed effects of multilevel linear mixed‐effects models for log_10_ heart rate, log_10_ stroke volume and log_10_ cardiac output.
**Table S3** Fixed effects of multilevel linear mixed‐effects models for log_10_ systemic vascular resistance and log_10_ mean arterial pressure.
**Table S4** Estimated marginal means (antilog values) for maternal hemodynamic variables, derived from multilevel linear mixed‐effects models, across clinical visits and study groups, showing significance levels for group comparisons.
**Table S5** Estimated marginal mean differences (95% CI), Cohen's *d* effect sizes (95% CI) and minimal detectable differences for hemodynamic variables, with *P*‐values for pairwise group comparisons.
**Table S6** Linear mixed‐effects models excluding participants with chronic hypertension.
**Table S7** Number of cases per study group included in analysis at each clinical visit.
**Table S8** Sensitivity analysis of mixed‐effects models for hemodynamic variables.

## Data Availability

Research data are not shared.
