# Supplementary material for: Longitudinal maternal hemodynamics in high‐risk pregnancies with different subtypes of pre‐eclampsia
Source: Ultrasound Obstet Gynecol. 2026 Apr 10;67(5):623–34. doi: 10.1002/uog.70215 (PMC13136059; doi:10.1002/uog.70215)
Supplement: Supplementary file 1 — Appendix S1 Mixed‐effects model selection. Table S1 Comparison of baseline maternal characteristics between included and excluded cases. Table S2 Fixed effects of multilevel linear mixed‐effects models for log10 heart rate, log10 stroke volume and log10 cardiac output. Table S3 Fixed effects of multilevel linear mixed‐effects models for log10 systemic vascular resistance and log10 mean arterial pressure. Table S4 Estimated marginal means (antilog values) for maternal hemodynamic variables, derived from multilevel linear mixed‐effects models, across clinical visits and study groups, showing significance levels for group comparisons. Table S5 Estimated marginal mean differences (95% CI), Cohen's d effect sizes (95% CI) and minimal detectable differences for hemodynamic variables, with P‐values for pairwise group comparisons. Table S6 Linear mixed‐effects models excluding participants with chronic hypertension. Table S7 Number of cases per study group included in analysis at each clinical visit. Table S8 Sensitivity analysis of mixed‐effects models for hemodynamic variables. [file UOG-67-623-s001.docx]

**Page 2: Appendix S1.** Mixed-effects model selection.

**Page 3: Table S1.** Comparison of baseline maternal characteristics between included and excluded cases.

**Page 4: Table S2**. Fixed effects of multilevel linear mixed-effects models for log_10_ heart rate, log_10_ stroke volume and log_10_ cardiac output.

**Page 6: Table S3.** Fixed effects of multilevel linear mixed-effects models for log_10_ systemic vascular resistance and log_10_ mean arterial pressure.

**Page 8: Table S4.** Estimated marginal means (antilog values) for maternal hemodynamic variables, derived from multilevel linear mixed-effects models, across clinical visits and study groups, showing significance levels for group comparisons.

**Page 10: Table S5.** Estimated marginal mean differences (95% CI), Cohen’s *d* effect sizes (95% CI) and minimal detectable differences for hemodynamic variables, with *P*-values for pairwise group comparisons.

**Page 16: Table S6.** Linear mixed-effects models excluding participants with chronic hypertension.

**Page 18: Table S7.** Number of cases per study group included in analysis at each clinical visit.

**Page 19: Table S8.** Sensitivity analysis of mixed-effects models for hemodynamic variables.

**Appendix S1** Mixed-effects model selection.

Mixed-effects model selection of log_10_ heart rate

A mixed-effects model analysis showed that the random intercept–random slope model (Akaike information criterion [AIC] = -9490.6) provided a better fit to the data than the fixed intercept–fixed slope model (AIC = -8827.4), the fixed intercept–random slope model (AIC = -9185.1), or the random intercept–fixed slope model (AIC = -9447.8).

Mixed-effects model selection of log_10_ stroke volume

A mixed-effects model analysis showed that the random intercept–random slope model (AIC = -5950.1) provided a better fit to the data than the fixed intercept–fixed slope model (AIC = -5473.2), the fixed intercept–random slope model (AIC = -5758.3), or the random intercept–fixed slope model (AIC = -5948.5).

Mixed-effects model selection of log_10_ cardiac output

A mixed-effects model analysis showed that the random intercept–random slope model (AIC = -5310.1) provided a better fit to the data than the fixed intercept–fixed slope model (AIC = -4944.9), the fixed intercept–random slope model (AIC = -5147.3), or the random intercept–fixed slope model (AIC = -5306.8).

Mixed-effects model selection of log_10_ systemic vascular resistance

A mixed-effects model analysis showed that the random intercept–random slope model (AIC = -5121.8) provided a better fit to the data than the fixed intercept–fixed slope model (AIC = -4768.7), the fixed intercept–random slope model (AIC = -4980.3), or the random intercept–fixed slope model (AIC = -5121.8).

Mixed-effects model selection of log_10_ mean arterial pressure

A mixed-effects model analysis showed that the random intercept–random slope model (AIC = -11898) provided a better fit to the data than the fixed intercept–fixed slope model (AIC = -11183), the fixed intercept–random slope model (AIC = -11538), or the random intercept–fixed slope model (AIC = -11856).

**Table S1** Comparison of baseline maternal characteristics between included and excluded cases.

| Variable | **Included cases**  **(n=1078)** | **Excluded cases**  **(n=68)** | **Overall**  **p-value** |
| --- | --- | --- | --- |
| Maternal age, years | 34.1 (31.5–37.1) | 34.8 (32.1–37.7) | 0.262 |
| Height, cm | 159 (156–162) | 159 (155–163) | 0.872 |
| Weight, kg | 57.3 (51.7–64.4) | 56.8 (51.3–63.8) | 0.774 |
| Smoking at conception | 72 (6.7%) | 1 (1.5%) | 0.120 |
| Method of conception |  |  | 0.027 |
| Spontaneous | 964 (89.4%) | 54 (79.4%) |  |
| In vitro fertilization | 96 (8.9%) | 13 (19.1%) |  |
| Ovulation induction | 18 (1.7%) | 1 (1.5%) |  |
| Parity |  |  | 0.627 |
| Nulliparous | 741 (68.7%) | 46 (67.6%) |  |
| Parous with previous history of preeclampsia | 283 (26.3%) | 17 (25.0%) |  |
| Parous without previous history of preeclampsia | 54 (5.0%) | 5 (7.4%) |  |
| Chronic hypertension | 32 (3.0%) | 4 (5.9%) | 0.160 |
| Diabetes mellitus | 16 (1.5%) | 2 (2.9%) | 0.290 |
| Antiphospholipid syndrome or systemic lupus erythematosus | 9 (0.8%) | 1 (1.5%) | 0.459 |
| Family history of preeclampsia | 15 (1.4%) | 2 (2.9%) | 0.267 |
| Gestational age at screening, days | 87 (85–89) | 87 (84–89) | 0.934 |
| CRL at screening, mm | 59.1 (55.3–63.5) | 59.7 (54.8–63.9) | 0.862 |
| MAP MoM | 1.067 (0.993–1.142) | 1.066 (0.994–1.134) | 0.999 |
| UtA-PI MoM | 1.107 (0.920–1.296) | 1.051 (0.842–1.298) | 0.453 |
| PlGF MoM | 0.722 (0.491–1.035) | 0.625 (0.433–1.038) | 0.260 |
| Background PE risk (1:n) | 185 (124–280) | 165 (90–237) | 0.049 |
| Adjusted PE risk (1:n) | 75 (34–392) | 64 (24–227) | 0.106 |

Values are presented as median (interquartile range) or number (%).

**Table S2** Fixed effects of multilevel linear mixed-effects models for log_10_ heart rate, log_10_ stroke volume and log_10_ cardiac output.

| Parameter | Log_10_ Heart rate | | | | Log_10_ Stroke volume | | Log_10_ Cardiac output | | | |
| --- | --- | --- | --- | --- | --- | --- | --- | --- | --- | --- |
| Fixed part | Estimate | Standard error | p-value | Estimate | Standard error | p-value | Estimate | Standard error | p-value |  |
| Intercept | 1.86800 | 0.00292 | <0.001 | 1.36200 | 0.07010 | <0.001 | 0.43770 | 0.07431 | <0.001 |  |
| Group (reference: group 1) |  |  |  |  |  |  |  |  |  |  |
| Group 2 | 0.01087 | 0.00380 | 0.004 | -0.04738 | 0.00658 | <0.001 | -0.03894 | 0.00725 | <0.001 |  |
| Group 3 | 0.00579 | 0.01136 | 0.610 | -0.05397 | 0.01934 | 0.005 | -0.05035 | 0.02133 | 0.018 |  |
| Group 4 | -0.00006 | 0.00948 | 0.995 | -0.11390 | 0.01650 | <0.001 | -0.11910 | 0.01815 | <0.001 |  |
| Visit (reference visit 1) |  |  |  |  |  |  |  |  |  |  |
| Visit 2 | 0.01207 | 0.00281 | <0.001 | 0.01366 | 0.00523 | 0.009 | 0.02417 | 0.00594 | <0.001 |  |
| Visit 3 | 0.01946 | 0.00320 | <0.001 | -0.00712 | 0.00602 | 0.237 | 0.00834 | 0.00683 | 0.222 |  |
| Interaction groups with Clinical visit |  |  |  |  |  |  |  |  |  |  |
| Group 2 * Visit 2 | -0.00164 | 0.00365 | 0.653 | -0.00198 | 0.00671 | 0.768 | -0.00347 | 0.00763 | 0.649 |  |
| Group 3 * Visit 2 | 0.01242 | 0.01085 | 0.252 | 0.00435 | 0.01998 | 0.828 | 0.01712 | 0.02270 | 0.451 |  |
| Group 4 * Visit 2 | 0.01032 | 0.00896 | 0.249 | 0.02073 | 0.01650 | 0.209 | 0.03146 | 0.01874 | 0.093 |  |
| Group 2 * Visit 3 | 0.00330 | 0.00414 | 0.426 | 0.01370 | 0.00736 | 0.063 | 0.01696 | 0.00839 | 0.043 |  |
| Group 3 * Visit 3 | 0.00021 | 0.01233 | 0.986 | 0.02211 | 0.02189 | 0.313 | 0.02301 | 0.02495 | 0.357 |  |
| Group 4 * Visit 3 | -0.04545 | 0.01272 | <0.001 | 0.05877 | 0.02265 | 0.010 | 0.01363 | 0.02564 | 0.595 |  |
| Maternal age (years) |  |  |  |  |  |  |  |  |  |  |
| Height (cm) |  |  |  | 0.00249 | 0.00045 | <0.001 | 0.00101 | 0.00048 | 0.037 |  |
| Weight (kg) |  |  |  | 0.00121 | 0.00023 | <0.001 | 0.00168 | 0.00025 | <0.001 |  |
| Smoking (reference non-smokers) |  |  |  |  |  |  |  |  |  |  |
| Conception (reference spontaneous) |  |  |  |  |  |  |  |  |  |  |
| In vitro fertilization |  |  |  |  |  |  |  |  |  |  |
| Ovulation induction |  |  |  |  |  |  |  |  |  |  |
| Parity (reference nulliparous) |  |  |  |  |  |  |  |  |  |  |
| Parous, no previous PE |  |  |  | 0.01547 | 0.00562 | 0.006 | 0.02305 | 0.00595 | <0.001 |  |
| Parous, previous PE |  |  |  | 0.02237 | 0.01166 | 0.055 | 0.03059 | 0.01237 | 0.014 |  |
| Chronic hypertension | 0.02230 | 0.00883 | 0.012 | 0.03582 | 0.01488 | 0.016 | 0.05416 | 0.01579 | <0.001 |  |
| Diabetes mellitus |  |  |  |  |  |  |  |  |  |  |
| Antiphospholipid syndrome or systemic lupus erythematosus |  |  |  |  |  |  |  |  |  |  |
| Family history of PE |  |  |  |  |  |  |  |  |  |  |

PE, preeclampsia.

There was no significant contribution from maternal age, height, weight, smoking, conception, parity, diabetes mellitus, antiphospholipid syndrome/systemic lupus erythematosus, and family history of PE on log_10_ heart rate.

There was no significant contribution from maternal age, smoking, conception, diabetes mellitus, antiphospholipid syndrome/systemic lupus erythematosus, and family history of PE on log_10_ stroke volume.

There was no significant contribution from maternal age, smoking, conception, diabetes mellitus, antiphospholipid syndrome/systemic lupus erythematosus, and family history of PE on log_10_ cardiac output.

**Table S3** Fixed effects of multilevel linear mixed-effects models for log_10_ systemic vascular resistance and log_10_ mean arterial pressure.

| Parameter | Log_10_ Systemic vascular resistance | | | Log_10_ Mean arterial pressure | | |
| --- | --- | --- | --- | --- | --- | --- |
| Fixed part | Estimate | Standard error | p-value | Estimate | Standard error | p-value |
| Intercept | 3.33500 | 0.07539 | <0.001 | 1.85800 | 0.02854 | <0.001 |
| Group (reference: group 1) |  |  |  |  |  |  |
| Group 2 | 0.07538 | 0.00734 | <0.001 | 0.03809 | 0.00267 | <0.001 |
| Group 3 | 0.09990 | 0.02168 | <0.001 | 0.05196 | 0.00787 | <0.001 |
| Group 4 | 0.17910 | 0.01829 | <0.001 | 0.06477 | 0.00659 | <0.001 |
| Visit (reference visit 1) |  |  |  |  |  |  |
| Visit 2 | -0.04022 | 0.00607 | <0.001 | -0.01572 | 0.00187 | <0.001 |
| Visit 3 | -0.01769 | 0.00674 | 0.009 | -0.00854 | 0.00234 | <0.001 |
| Interaction groups with time |  |  |  |  |  |  |
| Group 2 * Visit 2 | 0.00033 | 0.00787 | 0.967 | -0.00323 | 0.00239 | 0.177 |
| Group 3 * Visit 2 | -0.01591 | 0.02342 | 0.497 | 0.00109 | 0.00712 | 0.879 |
| Group 4 * Visit 2 | -0.02621 | 0.01934 | 0.175 | 0.00511 | 0.00588 | 0.385 |
| Group 2 * Visit 3 | -0.02172 | 0.00873 | 0.013 | -0.00479 | 0.00286 | 0.095 |
| Group 3 * Visit 3 | -0.01364 | 0.02595 | 0.599 | 0.00890 | 0.00852 | 0.296 |
| Group 4 * Visit 3 | 0.00900 | 0.02667 | 0.736 | 0.02379 | 0.00868 | 0.006 |
| Maternal age (years) |  |  |  |  |  |  |
| Height (cm) | -0.00149 | 0.00047 | 0.002 | -0.00038 | 0.00018 | 0.042 |
| Weight (kg) |  |  |  | 0.00160 | 0.00009 | <0.001 |
| Smoking (reference non-smokers) |  |  |  | -0.01220 | 0.00395 | 0.002 |
| Conception (reference spontaneous) |  |  |  |  |  |  |
| In vitro fertilization |  |  |  |  |  |  |
| Ovulation induction |  |  |  |  |  |  |
| Parity (reference nulliparous) |  |  |  |  |  |  |
| Parous, no previous PE | -0.02172 | 0.00606 | <0.001 |  |  |  |
| Parous, previous PE | -0.02419 | 0.01258 | 0.055 |  |  |  |
| Chronic hypertension |  |  |  | 0.03830 | 0.00604 | <0.001 |
| Diabetes mellitus |  |  |  |  |  |  |
| Antiphospholipid syndrome or systemic lupus erythematosus |  |  |  |  |  |  |
| Family history of PE |  |  |  |  |  |  |

PE, preeclampsia.

There was no significant contribution from maternal age, weight, smoking, conception, chronic hypertension, diabetes mellitus, antiphospholipid syndrome/systemic lupus erythematosus, and family history of PE on log_10_ systemic vascular resistance.

There was no significant contribution from maternal age, conception, parity, diabetes mellitus, antiphospholipid syndrome/systemic lupus erythematosus, and family history of PE on log_10_ mean arterial pressure

**Table S4.** Estimated marginal means (antilog values) for maternal hemodynamic variables, derived from multilevel linear mixed-effects models, across clinical visits and study groups, showing significance levels for group comparisons.

| **Hemodynamic variable** | | | **Group 1: low risk without PE (*n* = 407)** | **Group 2: high risk without PE (*n* = 598)** | **Group 3: high risk with term PE (*n* = 29)** | **Group 4: high risk with preterm PE (*n* = 44)** | ***P* (between groups)** | ***P* (throughout pregnancy)** |
| --- | --- | --- | --- | --- | --- | --- | --- | --- |
| Heart rate | | |  |  |  |  |  | 0.0002^d**,h*^ |
| Visit 1 (bpm) | | 75.68 (73.89–77.51) | 77.60 (75.95–79.29) | 76.70 (72.82–80.78) | 75.67 (72.55–78.92) | 0.0332^d*^ |  |  |
| Visit 2 (bpm) | | 77.81 (75.99–79.68) | 79.48 (77.81–81.20) | 81.15 (77.17–85.33) | 79.67 (76.49–82.99) | 0.0495 |  |  |
| Visit 3 (bpm) | | 79.15 (77.25–81.09) | 81.78 (80.02–83.57) | 80.25 (76.08–84.65) | 71.27 (67.43–75.34) | <0.0001^d**,f**,h****,i**^ |  |  |
| *P* (between visits) | | <0.0001^a****,b****,c*^ | <0.0001^a****,b****,c****^ | 0.0583 | 0.0001^a*,b*,c***^ |  |  |  |
| Stroke volume | | |  |  |  |  |  | <0.0001  ^d****,e**,f****,h**^ |
| Visit 1 (mL) | 73.29 (70.26–76.44) | 65.71 (63.27–68.25) | 64.72 (59.24–70.72) | 56.38 (52.48–60.57) | <0.0001^d****,e*, f****,h***,i*^ |  |  |  |
| Visit 2 (mL) | 75.63 (72.51–78.88) | 67.50 (64.98–70.13) | 67.46 (61.84–73.60) | 61.03 (56.87–65.49) | <0.0001^d****,e*,f****,h*^ |  |  |  |
| Visit 3 (mL) | 72.10 (69.01–75.32) | 66.72 (64.10–69.44) | 67.00 (61.06–73.52) | 63.50 (57.60–70.01) | <0.0001^d****^ |  |  |  |
| *P* (between visits) | 0.0004^a*,c***^ | 0.0263^a*^ | 0.6249 | 0.0251 |  |  |  |  |
| Cardiac output | | |  |  |  |  |  | <0.0001 ^d****,e*,f****,h****,i**^ |
| Visit 1 (L/min) | 5.602 (5.354–5.861) | 5.121 (4.918–5.334) | 4.989 (4.526–5.498) | 4.258 (3.936–4.607) | <0.0001 ^d****,e*,f****,h****,i*^ |  |  |  |
| Visit 2 (L/min) | 5.922 (5.662–6.195) | 5.371 (5.157–5.594) | 5.486 (4.992–6.029) | 4.840 (4.484–5.225) | <0.0001^d****,f****,h*^ |  |  |  |
| Visit 3 (L/min) | 5.710 (5.450–5.983) | 5.429 (5.202–5.665) | 5.362 (4.851–5.927) | 4.479 (4.026–4.984) | <0.0001^d*,f***,h**,i*^ |  |  |  |
| *P* (between visits) | 0.0002^a***, c*^ | <0.0001^a***, b****^ | 0.1586 | 0.0071^a**^ |  |  |  |  |
| SVR | | |  |  |  |  |  | <0.0001 ^d****,e****,f****,h****,i***^ |
| Visit 1 (dyn × s × cm^−5^) | 1208.2 (1171.1–1246.5) | 1437.2 (1399.4–1476.0) | 1520.7 (1382.3–1672.9) | 1824.7 (1689.8–1970.34) | <0.0001 ^d****,e****,f****,h****,i**^ |  |  |  |
| Visit 2 (dyn × s × cm^−5^) | 1101.3 (1068.5–1135.6) | 1311.1 (1277.1–1345.9) | 1336.3 (1218.2–1465.8) | 1565.8 (1453.6–1686.8) | <0.0001 ^d****,e***,f****,h****,i*^ |  |  |  |
| Visit 3 (dyn × s × cm^−5^) | 1160.0 (1123.1–1198.0) | 1312.5 (1277.1–1348.9) | 1414.8 (1281.0–1562.6) | 1788.5 (1606.2–1991.6) | <0.0001 ^d****,e***,f****,h****,i**^ |  |  |  |
| *P* (between visits) | <0.001 ^a****,b**,c***^ | <0.001 ^a****,b****^ | 0.046 ^a*^ | <0.001 ^a***,c*^ |  |  |  |  |
| MAP | | |  |  |  |  |  | <0.0001 ^d****,e****,f****,g**,h****,i*^ |
| Visit 1 (mmHg) | 81.52 (80.03–83.04) | 88.99 (87.53–90.47) | 91.88 (88.55–95.33) | 94.63 (91.78–97.56) | <0.0001 ^d****,e****,f****,h***^ |  |  |  |
| Visit 2 (mmHg) | 78.62 (77.20–80.06) | 85.19 (83.81–86.59) | 88.83 (85.81–91.96) | 92.34 (89.72–95.04) | <0.0001 ^d****,e****,f****,g*,h****^ |  |  |  |
| Visit 3 (mmHg) | 79.93 (78.45–81.44) | 86.30 (84.85–87.78) | 91.95 (88.63–95.41) | 98.01 (94.33–101.83) | <0.0001 ^d****,e****,f****, g***,h****,i*^ |  |  |  |
| *P* (between visits) | <0.0001 ^a****,b***,c***^ | <0.0001 ^a****,b****,c***^ | 0.0344 | 0.0020 ^c**^ |  |  |  |  |

Comparisons are denoted by lowercase letters: a, visit 1 vs visit 2; b, visit 1 vs visit 3; c, visit 2 vs visit 3; d, group 1 vs group 2; e, group 1 vs group 3; f, group 1 vs group 4; g, group 2 vs group 3; h, group 2 vs group 4; i, group 3 vs group 4. Significance levels are shown as *P < 0.05, **P < 0.01, ***P < 0.001, ****P<0.0001.

**Table S5**. Estimated marginal mean differences (95% CI), Cohen’s *d* effect sizes (95% CI) and minimal detectable differences for hemodynamic variables, with *P*-values for pairwise group comparisons.

| Timepoint | Groups comparison | Estimated marginal mean difference | | | Minimal detectable difference | Effect size (Cohen’s d) | | | Holm-Bonferroni P-value |
| --- | --- | --- | --- | --- | --- | --- | --- | --- | --- |
|  |  | Estimated marginal mean difference | Lower 95% CI | Upper 95%CI |  | Effect size | Lower 95% CI | Upper 95%CI |  |
| Log_10_ Heart rate |  |  |  |  |  |  |  |  |  |
| Throughout gestation | Group 1 - Group 2 | -0.01142 | -0.01958 | -0.00327 | 0.00864 | -0.29657 | -0.45350 | -0.13964 | 0.0013 |
|  | Group 1 - Group 3 | -0.01000 | -0.03434 | 0.01434 | 0.02580 | -0.25962 | -0.72810 | 0.20887 | 0.5553 |
|  | Group 1 - Group 4 | 0.01177 | -0.00972 | 0.03327 | 0.02279 | 0.30559 | -0.10829 | 0.71948 | 0.4443 |
|  | Group 2 - Group 3 | 0.00142 | -0.02259 | 0.02543 | 0.02545 | 0.03696 | -0.42520 | 0.49911 | 0.8755 |
|  | Group 2 - Group 4 | 0.02320 | 0.00214 | 0.04425 | 0.02232 | 0.60217 | 0.19674 | 1.00759 | 0.0183 |
|  | Group 3 - Group 4 | 0.02177 | -0.00914 | 0.05269 | 0.03277 | 0.56521 | -0.02995 | 1.16037 | 0.2518 |
| Visit 1 (12^+0^–15^+6^ weeks) | Group 1 - Group 2 | -0.01087 | -0.02091 | -0.00083 | 0.01064 | -0.28217 | -0.47539 | -0.08896 | 0.0257 |
|  | Group 1 - Group 3 | -0.00579 | -0.03580 | 0.02422 | 0.03182 | -0.15031 | -0.72815 | 0.42753 | 1.0000 |
|  | Group 1 - Group 4 | 0.00006 | -0.02500 | 0.02512 | 0.02657 | 0.00161 | -0.48093 | 0.48415 | 1.0000 |
|  | Group 2 - Group 3 | 0.00508 | -0.02456 | 0.03472 | 0.03142 | 0.13186 | -0.43878 | 0.70250 | 1.0000 |
|  | Group 2 - Group 4 | 0.01093 | -0.01362 | 0.03548 | 0.02603 | 0.28378 | -0.18894 | 0.75650 | 1.0000 |
|  | Group 3 - Group 4 | 0.00585 | -0.03142 | 0.04313 | 0.03952 | 0.15192 | -0.56573 | 0.86956 | 1.0000 |
| Visit 2 (20^+0^–24^+6^ weeks) | Group 1 - Group 2 | -0.00923 | -0.01896 | 0.00050 | 0.01032 | -0.23964 | -0.42713 | -0.05216 | 0.0739 |
|  | Group 1 - Group 3 | -0.01821 | -0.04717 | 0.01075 | 0.03072 | -0.47278 | -1.03067 | 0.08512 | 0.4845 |
|  | Group 1 - Group 4 | -0.01026 | -0.03447 | 0.01395 | 0.02568 | -0.26636 | -0.73269 | 0.19997 | 1.0000 |
|  | Group 2 - Group 3 | -0.00898 | -0.03758 | 0.01961 | 0.03033 | -0.23313 | -0.78400 | 0.31773 | 1.0000 |
|  | Group 2 - Group 4 | -0.00103 | -0.02474 | 0.02268 | 0.02515 | -0.02671 | -0.48338 | 0.42995 | 1.0000 |
|  | Group 3 - Group 4 | 0.00795 | -0.02800 | 0.04390 | 0.03814 | 0.20642 | -0.48614 | 0.89898 | 1.0000 |
| Visit 3 (30^+0^–37^+6^ weeks) | Group 1 - Group 2 | -0.01417 | -0.02460 | -0.00374 | 0.01106 | -0.36790 | -0.56874 | -0.16707 | 0.0014 |
|  | Group 1 - Group 3 | -0.00600 | -0.03701 | 0.02501 | 0.03287 | -0.15576 | -0.75275 | 0.44124 | 0.9612 |
|  | Group 1 - Group 4 | 0.04552 | 0.01275 | 0.07828 | 0.03475 | 1.18153 | 0.55045 | 1.81261 | 0.0013 |
|  | Group 2 - Group 3 | 0.00817 | -0.02244 | 0.03878 | 0.03245 | 0.21215 | -0.37719 | 0.80148 | 0.9612 |
|  | Group 2 - Group 4 | 0.05969 | 0.02734 | 0.09204 | 0.03431 | 1.54944 | 0.92632 | 2.17256 | <0.0001 |
|  | Group 3 - Group 4 | 0.05152 | 0.00809 | 0.09494 | 0.04605 | 1.33729 | 0.50101 | 2.17357 | 0.0053 |
| Log_10_ Stroke volume |  |  |  |  |  |  |  |  |  |
| Throughout gestation | Group 1 - Group 2 | 0.04347 | 0.02954 | 0.05741 | 0.01477 | 0.60393 | 0.46038 | 0.74748 | <0.0001 |
|  | Group 1 - Group 3 | 0.04515 | 0.00465 | 0.08564 | 0.04292 | 0.62720 | 0.21007 | 1.04433 | 0.0098 |
|  | Group 1 - Group 4 | 0.08739 | 0.05016 | 0.12462 | 0.03947 | 1.21396 | 0.83035 | 1.59758 | <0.0001 |
|  | Group 2 - Group 3 | 0.00167 | -0.03807 | 0.04142 | 0.04213 | 0.02327 | -0.38616 | 0.43270 | 0.9113 |
|  | Group 2 - Group 4 | 0.04391 | 0.00788 | 0.07995 | 0.03821 | 0.61003 | 0.23870 | 0.98136 | 0.0053 |
|  | Group 3 - Group 4 | 0.04224 | -0.00953 | 0.09400 | 0.05488 | 0.58676 | 0.05344 | 1.12009 | 0.0625 |
| Visit 1 (12^+0^–15^+6^ weeks) | Group 1 - Group 2 | 0.04738 | 0.02999 | 0.06478 | 0.01844 | 0.65822 | 0.47899 | 0.83744 | <0.0001 |
|  | Group 1 - Group 3 | 0.05397 | 0.00287 | 0.10507 | 0.05418 | 0.74969 | 0.22317 | 1.27622 | 0.0160 |
|  | Group 1 - Group 4 | 0.11389 | 0.07030 | 0.15748 | 0.04622 | 1.58209 | 1.13291 | 2.03127 | <0.0001 |
|  | Group 2 - Group 3 | 0.00659 | -0.04372 | 0.05689 | 0.05333 | 0.09148 | -0.42682 | 0.60978 | 0.7295 |
|  | Group 2 - Group 4 | 0.06651 | 0.02418 | 0.10883 | 0.04487 | 0.92387 | 0.48778 | 1.35997 | 0.0001 |
|  | Group 3 - Group 4 | 0.05992 | -0.00361 | 0.12345 | 0.06736 | 0.83239 | 0.17776 | 1.48703 | 0.0256 |
| Visit 2 (20^+0^–24^+6^ weeks) | Group 1 - Group 2 | 0.04936 | 0.03225 | 0.06647 | 0.01815 | 0.68570 | 0.50935 | 0.86204 | <0.0001 |
|  | Group 1 - Group 3 | 0.04962 | -0.00039 | 0.09963 | 0.05305 | 0.68932 | 0.17376 | 1.20489 | 0.0265 |
|  | Group 1 - Group 4 | 0.09316 | 0.05046 | 0.13586 | 0.04530 | 1.29414 | 0.85390 | 1.73439 | <0.0001 |
|  | Group 2 - Group 3 | 0.00026 | -0.04898 | 0.04950 | 0.05223 | 0.00363 | -0.50399 | 0.51125 | 0.9888 |
|  | Group 2 - Group 4 | 0.04380 | 0.00233 | 0.08527 | 0.04399 | 0.60845 | 0.18093 | 1.03597 | 0.0213 |
|  | Group 3 - Group 4 | 0.04354 | -0.01865 | 0.10572 | 0.06596 | 0.60482 | -0.03626 | 1.24590 | 0.1292 |
| Visit 3 (30^+0^–37^+6^ weeks) | Group 1 - Group 2 | 0.03368 | 0.01544 | 0.05192 | 0.01934 | 0.46788 | 0.27991 | 0.65586 | <0.0001 |
|  | Group 1 - Group 3 | 0.03186 | -0.02154 | 0.08526 | 0.05661 | 0.44258 | -0.10759 | 0.99274 | 0.4605 |
|  | Group 1 - Group 4 | 0.05512 | -0.00302 | 0.11325 | 0.06165 | 0.76566 | 0.16649 | 1.36483 | 0.0618 |
|  | Group 2 - Group 3 | -0.00182 | -0.05438 | 0.05074 | 0.05572 | -0.02530 | -0.56683 | 0.51622 | 0.9653 |
|  | Group 2 - Group 4 | 0.02144 | -0.03570 | 0.07857 | 0.06059 | 0.29778 | -0.29107 | 0.88663 | 0.9653 |
|  | Group 3 - Group 4 | 0.02326 | -0.05233 | 0.09885 | 0.08016 | 0.32308 | -0.45592 | 1.10209 | 0.9653 |
| Log_10_ Cardiac output |  |  |  |  |  |  |  |  |  |
| Throughout gestation | Group 1 - Group 2 | 0.03444 | 0.01968 | 0.04920 | 0.01565 | 0.42179 | 0.28772 | 0.55586 | <0.0001 |
|  | Group 1 - Group 3 | 0.03698 | -0.00590 | 0.07985 | 0.04544 | 0.45286 | 0.06351 | 0.84221 | 0.0457 |
|  | Group 1 - Group 4 | 0.10404 | 0.06437 | 0.14372 | 0.04207 | 1.27427 | 0.91383 | 1.63472 | <0.0001 |
|  | Group 2 - Group 3 | 0.00254 | -0.03954 | 0.04461 | 0.04460 | 0.03107 | -0.35104 | 0.41318 | 0.8734 |
|  | Group 2 - Group 4 | 0.06960 | 0.03119 | 0.10801 | 0.04073 | 0.85248 | 0.50353 | 1.20144 | <0.0001 |
|  | Group 3 - Group 4 | 0.06707 | 0.01209 | 0.12204 | 0.05828 | 0.82141 | 0.32206 | 1.32076 | 0.0039 |
| Visit 1 (12^+0^–15^+6^ weeks) | Group 1 - Group 2 | 0.03894 | 0.01978 | 0.05810 | 0.02032 | 0.47688 | 0.30281 | 0.65095 | <0.0001 |
|  | Group 1 - Group 3 | 0.05035 | -0.00600 | 0.10670 | 0.05975 | 0.61668 | 0.10473 | 1.12863 | 0.0368 |
|  | Group 1 - Group 4 | 0.11907 | 0.07111 | 0.16703 | 0.05086 | 1.45833 | 1.02257 | 1.89409 | <0.0001 |
|  | Group 2 - Group 3 | 0.01141 | -0.04408 | 0.06691 | 0.05883 | 0.13980 | -0.36431 | 0.64392 | 0.5868 |
|  | Group 2 - Group 4 | 0.08013 | 0.03353 | 0.12674 | 0.04942 | 0.98146 | 0.55803 | 1.40488 | <0.0001 |
|  | Group 3 - Group 4 | 0.06872 | -0.00135 | 0.13878 | 0.07429 | 0.84165 | 0.20513 | 1.47817 | 0.0290 |
| Visit 2 (20^+0^–24^+6^ weeks) | Group 1 - Group 2 | 0.04241 | 0.02384 | 0.06097 | 0.01969 | 0.51939 | 0.35065 | 0.68813 | <0.0001 |
|  | Group 1 - Group 3 | 0.03323 | -0.02105 | 0.08752 | 0.05759 | 0.40703 | -0.08643 | 0.90049 | 0.2122 |
|  | Group 1 - Group 4 | 0.08761 | 0.04132 | 0.13391 | 0.04911 | 1.07306 | 0.65227 | 1.49384 | <0.0001 |
|  | Group 2 - Group 3 | -0.00917 | -0.06263 | 0.04429 | 0.05671 | 0.11235 | -0.59828 | 0.37358 | 0.6505 |
|  | Group 2 - Group 4 | 0.04521 | 0.00023 | 0.09018 | 0.04771 | 0.55367 | 0.14485 | 0.96249 | 0.0320 |
|  | Group 3 - Group 4 | 0.05438 | -0.01312 | 0.12188 | 0.07161 | 0.66603 | 0.05247 | 1.27958 | 0.1005 |
| Visit 3 (30^+0^–37^+6^ weeks) | Group 1 - Group 2 | 0.02197 | 0.00227 | 0.04168 | 0.02089 | 0.26910 | 0.09008 | 0.44812 | 0.0131 |
|  | Group 1 - Group 3 | 0.02734 | -0.03034 | 0.08502 | 0.06115 | 0.33487 | -0.18910 | 0.85883 | 0.4212 |
|  | Group 1 - Group 4 | 0.10544 | 0.04181 | 0.16908 | 0.06748 | 1.29143 | 0.71320 | 1.86966 | 0.0001 |
|  | Group 2 - Group 3 | 0.00537 | -0.05141 | 0.06215 | 0.06019 | 0.06576 | -0.45001 | 0.58153 | 0.8027 |
|  | Group 2 - Group 4 | 0.08347 | 0.02089 | 0.14605 | 0.06636 | 1.02232 | 0.45369 | 1.59096 | 0.0022 |
|  | Group 3 - Group 4 | 0.07810 | -0.00422 | 0.16042 | 0.08729 | 0.95656 | 0.20864 | 1.70449 | 0.0369 |
| Log_10_ Systemic vascular resistance |  |  |  |  |  |  |  |  |  |
| Throughout gestation | Group 1 - Group 2 | -0.06825 | -0.08319 | -0.05331 | 0.01584 | -0.81266 | -0.94458 | -0.68074 | <0.0001 |
|  | Group 1 - Group 3 | -0.09005 | -0.13369 | -0.04641 | 0.04626 | -1.07221 | -1.45751 | -0.68691 | <0.0001 |
|  | Group 1 - Group 4 | -0.17332 | -0.21343 | -0.13321 | 0.04253 | -2.06366 | -2.41791 | -1.70940 | <0.0001 |
|  | Group 2 - Group 3 | -0.02180 | -0.06482 | 0.02123 | 0.04560 | -0.25955 | -0.63941 | 0.12031 | 0.1808 |
|  | Group 2 - Group 4 | -0.10507 | -0.14418 | -0.06595 | 0.04147 | -1.25100 | -1.59646 | -0.90553 | <0.0001 |
|  | Group 3 - Group 4 | -0.08327 | -0.13963 | -0.02691 | 0.05975 | -0.99145 | -1.48912 | -0.49377 | 0.0002 |
| Visit 1 (12^+0^–15^+6^ weeks) | Group 1 - Group 2 | -0.07539 | -0.09478 | -0.05599 | 0.02056 | -0.89758 | -1.06887 | -0.72629 | <0.0001 |
|  | Group 1 - Group 3 | -0.09990 | -0.15718 | -0.04262 | 0.06073 | -1.18950 | -1.69538 | -0.68362 | <0.0001 |
|  | Group 1 - Group 4 | -0.17906 | -0.22739 | -0.13072 | 0.05125 | -2.13196 | -2.55890 | -1.70503 | <0.0001 |
|  | Group 2 - Group 3 | -0.02452 | -0.08107 | 0.03204 | 0.05996 | -0.29192 | -0.79139 | 0.20756 | 0.2522 |
|  | Group 2 - Group 4 | -0.10367 | -0.15091 | -0.05643 | 0.05009 | -1.23438 | -1.65161 | -0.81715 | <0.0001 |
|  | Group 3 - Group 4 | -0.07915 | -0.15060 | -0.00770 | 0.07575 | -0.94246 | -1.57350 | -0.31143 | 0.0070 |
| Visit 2 (20^+0^–24^+6^ weeks) | Group 1 - Group 2 | -0.07571 | -0.09463 | -0.05679 | 0.02007 | -0.90146 | -1.06863 | -0.73430 | <0.0001 |
|  | Group 1 - Group 3 | -0.08399 | -0.13949 | -0.02849 | 0.05888 | -1.00006 | -1.49051 | -0.50960 | 0.0002 |
|  | Group 1 - Group 4 | -0.15284 | -0.19975 | -0.10593 | 0.04977 | -1.81983 | -2.23438 | -1.40529 | <0.0001 |
|  | Group 2 - Group 3 | -0.00828 | -0.06307 | 0.04651 | 0.05812 | -0.09859 | -0.58276 | 0.38558 | 0.6898 |
|  | Group 2 - Group 4 | -0.07713 | -0.12296 | -0.03130 | 0.04861 | -0.91837 | -1.32333 | -0.51342 | <0.0001 |
|  | Group 3 - Group 4 | -0.06885 | -0.13807 | 0.00037 | 0.07343 | -0.81978 | -1.43143 | -0.20813 | 0.0174 |
| Visit 3 (30^+0^–37^+6^ weeks) | Group 1 - Group 2 | -0.05366 | -0.07401 | -0.03331 | 0.02157 | -0.63893 | -0.81863 | -0.45923 | <0.0001 |
|  | Group 1 - Group 3 | -0.08626 | -0.14601 | -0.02651 | 0.06334 | -1.02708 | -1.55470 | -0.49945 | 0.0004 |
|  | Group 1 - Group 4 | -0.18806 | -0.25396 | -0.12216 | 0.06989 | -2.23917 | -2.82134 | -1.65701 | <0.0001 |
|  | Group 2 - Group 3 | -0.03260 | -0.09158 | 0.02638 | 0.06253 | -0.38815 | -0.90899 | 0.13270 | 0.1444 |
|  | Group 2 - Group 4 | -0.13440 | -0.19936 | -0.06943 | 0.06890 | -1.60024 | -2.17414 | -1.02635 | <0.0001 |
|  | Group 3 - Group 4 | -0.10180 | -0.18744 | -0.01616 | 0.09081 | -1.21210 | -1.96852 | -0.45568 | 0.0034 |
| Log_10_ Mean arterial pressure |  |  |  |  |  |  |  |  |  |
| Throughout gestation | Group 1 - Group 2 | -0.03541 | -0.04103 | -0.02980 | 0.00595 | -1.43656 | -1.60543 | -1.26769 | <0.0001 |
|  | Group 1 - Group 3 | -0.05529 | -0.07174 | -0.03884 | 0.01743 | -2.24287 | -2.73762 | -1.74813 | <0.0001 |
|  | Group 1 - Group 4 | -0.07441 | -0.08895 | -0.05987 | 0.01541 | -3.01844 | -3.45591 | -2.58097 | <0.0001 |
|  | Group 2 - Group 3 | -0.01988 | -0.03609 | -0.00366 | 0.01719 | -0.80631 | -1.29414 | -0.31848 | 0.0025 |
|  | Group 2 - Group 4 | -0.03899 | -0.05322 | -0.02477 | 0.01508 | -1.58188 | -2.00982 | -1.15393 | <0.0001 |
|  | Group 3 - Group 4 | -0.01912 | -0.03996 | 0.00172 | 0.02209 | -0.77557 | -1.40263 | -0.14851 | 0.0155 |
| Visit 1 (12^+0^–15^+6^ weeks) | Group 1 - Group 2 | -0.03809 | -0.04513 | -0.03104 | 0.00747 | -1.54504 | -1.75697 | -1.33311 | <0.0001 |
|  | Group 1 - Group 3 | -0.05196 | -0.07277 | -0.03116 | 0.02206 | -2.10785 | -2.73383 | -1.48186 | <0.0001 |
|  | Group 1 - Group 4 | -0.06477 | -0.08218 | -0.04736 | 0.01846 | -2.62760 | -3.15142 | -2.10378 | <0.0001 |
|  | Group 2 - Group 3 | -0.01387 | -0.03441 | 0.00666 | 0.02177 | -0.56281 | -1.18075 | 0.05513 | 0.1490 |
|  | Group 2 - Group 4 | -0.02669 | -0.04372 | -0.00965 | 0.01806 | -1.08256 | -1.59507 | -0.57005 | 0.0001 |
|  | Group 3 - Group 4 | -0.01281 | -0.03863 | 0.01300 | 0.02737 | -0.51975 | -1.29646 | 0.25696 | 0.1899 |
| Visit 2 (20^+0^–24^+6^ weeks) | Group 1 - Group 2 | -0.03485 | -0.04141 | -0.02830 | 0.00695 | -1.41386 | -1.61122 | -1.21651 | <0.0001 |
|  | Group 1 - Group 3 | -0.05304 | -0.07230 | -0.03379 | 0.02043 | -2.15184 | -2.73153 | -1.57215 | <0.0001 |
|  | Group 1 - Group 4 | -0.06988 | -0.08602 | -0.05374 | 0.01712 | -2.83490 | -3.32081 | -2.34900 | <0.0001 |
|  | Group 2 - Group 3 | -0.01819 | -0.03720 | 0.00082 | 0.02016 | -0.73798 | -1.31022 | -0.16573 | 0.0231 |
|  | Group 2 - Group 4 | -0.03503 | -0.05082 | -0.01924 | 0.01675 | -1.42104 | -1.89635 | -0.94573 | <0.0001 |
|  | Group 3 - Group 4 | -0.01684 | -0.04072 | 0.00705 | 0.02534 | -0.68306 | -1.40210 | 0.03597 | 0.0628 |
| Visit 3 (30^+0^–37^+6^ weeks) | Group 1 - Group 2 | -0.03330 | -0.04030 | -0.02630 | 0.00742 | -1.35078 | -1.56142 | -1.14015 | <0.0001 |
|  | Group 1 - Group 3 | -0.06086 | -0.08142 | -0.04030 | 0.02180 | -2.46893 | -3.08759 | -1.85026 | <0.0001 |
|  | Group 1 - Group 4 | -0.08857 | -0.11034 | -0.06680 | 0.02309 | -3.59282 | -4.24805 | -2.93759 | <0.0001 |
|  | Group 2 - Group 3 | -0.02756 | -0.04785 | -0.00727 | 0.02151 | -1.11814 | -1.72865 | -0.50764 | 0.0007 |
|  | Group 2 - Group 4 | -0.05527 | -0.07676 | -0.03378 | 0.02279 | -2.24203 | -2.88879 | -1.59528 | <0.0001 |
|  | Group 3 - Group 4 | -0.02770 | -0.05649 | 0.00108 | 0.03053 | -1.12389 | -1.99022 | -0.25757 | 0.0111 |

**Table S6** Linear mixed-effects models excluding participants with chronic hypertension.

| **Parameter** | **Log_10_ Heart rate** | | | **Log_10_ Stroke volume** | | | **Log_10_ Cardiac output** | | |
| --- | --- | --- | --- | --- | --- | --- | --- | --- | --- |
| Fixed part | Estimate | Standard error | p-value | Estimate | Standard error | p-value | Estimate | Standard error | p-value |
| Intercept | 1.86783 | 0.00290 | <0.001 | 1.36922 | 0.07135 | <0.001 | 0.43845 | 0.07569 | <0.001 |
| Group (reference: group 1) |  |  |  |  |  |  |  |  |  |
| Group 2 | 0.01135 | 0.00378 | 0.003 | -0.04761 | 0.00662 | <0.001 | -0.03872 | 0.00730 | <0.001 |
| Group 3 | 0.00188 | 0.01182 | 0.874 | -0.05415 | 0.02030 | 0.008 | -0.05436 | 0.02240 | 0.015 |
| Group 4 | -0.00354 | 0.01016 | 0.728 | -0.09410 | 0.01779 | <0.001 | -0.10353 | 0.01960 | <0.001 |
| Visit (reference visit 1) |  |  |  |  |  |  |  |  |  |
| Visit 2 | 0.01207 | 0.00279 | <0.001 | 0.01359 | 0.00525 | 0.010 | 0.02404 | 0.00595 | <0.001 |
| Visit 3 | 0.01947 | 0.00315 | <0.001 | -0.00728 | 0.00604 | 0.228 | 0.00802 | 0.00682 | 0.240 |
| Interaction groups with Clinical visit |  |  |  |  |  |  |  |  |  |
| Group 2 * Visit 2 | -0.00234 | 0.00364 | 0.521 | -0.00235 | 0.00678 | 0.729 | -0.00455 | 0.00770 | 0.554 |
| Group 3 * Visit 2 | 0.01713 | 0.01132 | 0.131 | 0.00266 | 0.02108 | 0.900 | 0.02018 | 0.02393 | 0.399 |
| Group 4 * Visit 2 | 0.00850 | 0.00973 | 0.382 | 0.01011 | 0.01813 | 0.577 | 0.01901 | 0.02057 | 0.356 |
| Group 2 * Visit 3 | 0.00332 | 0.00411 | 0.418 | 0.01307 | 0.00742 | 0.079 | 0.01631 | 0.00842 | 0.053 |
| Group 3 * Visit 3 | 0.00459 | 0.01281 | 0.720 | 0.02583 | 0.02315 | 0.265 | 0.03122 | 0.02627 | 0.235 |
| Group 4 * Visit 3 | -0.04066 | 0.01358 | 0.003 | 0.04268 | 0.02457 | 0.083 | 0.00096 | 0.02772 | 0.972 |
| Height (cm) |  |  |  | 0.00244 | 0.00046 | <0.001 | 0.00099 | 0.00049 | 0.044 |
| Weight (kg) |  |  |  | 0.00123 | 0.00023 | <0.001 | 0.00172 | 0.00025 | <0.001 |
| Parity (reference nulliparous) |  |  |  |  |  |  |  |  |  |
| Parous, no previous PE |  |  |  | 0.01564 | 0.00569 | 0.006 | 0.02320 | 0.00604 | <0.001 |
| Parous, previous PE |  |  |  | 0.01735 | 0.01240 | 0.162 | 0.02810 | 0.01316 | 0.033 |
|  |  |  |  |  |  |  |  |  |  |
| **Parameter** | **Log_10_ Systemic vascular resistance** | | | **Log_10_ Mean arterial pressure** | | |  |  |  |
| Fixed part | Estimate | Standard error | p-value | Estimate | Standard error | p-value |  |  |  |
| Intercept | 3.32798 | 0.07704 | <0.001 | 1.85461 | 0.02882 | <0.001 |  |  |  |
| Group (reference: group 1) |  |  |  |  |  |  |  |  |  |
| Group 2 | 0.07527 | 0.00743 | <0.001 | 0.03776 | 0.00267 | <0.001 |  |  |  |
| Group 3 | 0.10589 | 0.02287 | <0.001 | 0.05215 | 0.00826 | <0.001 |  |  |  |
| Group 4 | 0.16772 | 0.02000 | <0.001 | 0.06640 | 0.00711 | <0.001 |  |  |  |
| Visit (reference visit 1) |  |  |  |  |  |  |  |  |  |
| Visit 2 | -0.04022 | 0.00609 | <0.001 | -0.01579 | 0.00186 | <0.001 |  |  |  |
| Visit 3 | -0.01769 | 0.00674 | 0.009 | -0.00871 | 0.00236 | <0.001 |  |  |  |
| Interaction groups with time |  |  |  |  |  |  |  |  |  |
| Group 2 * Visit 2 | 0.00172 | 0.00795 | 0.829 | -0.00293 | 0.00240 | 0.222 |  |  |  |
| Group 3 * Visit 2 | -0.02006 | 0.02473 | 0.417 | -0.00003 | 0.00747 | 0.997 |  |  |  |
| Group 4 * Visit 2 | -0.01154 | 0.02126 | 0.587 | 0.00733 | 0.00642 | 0.254 |  |  |  |
| Group 2 * Visit 3 | -0.02103 | 0.00879 | 0.017 | -0.00476 | 0.00290 | 0.101 |  |  |  |
| Group 3 * Visit 3 | -0.02229 | 0.02741 | 0.416 | 0.00831 | 0.00905 | 0.359 |  |  |  |
| Group 4 * Visit 3 | 0.02361 | 0.02891 | 0.414 | 0.02674 | 0.00944 | 0.005 |  |  |  |
| Height (cm) | -0.00145 | 0.00048 | 0.003 | -0.00036 | 0.00019 | 0.050 |  |  |  |
| Weight (kg) |  |  |  | 0.00162 | 0.00009 | <0.001 |  |  |  |
| Smoking (reference non-smokers) |  |  |  | -0.01170 | 0.00396 | 0.003 |  |  |  |
| Parity (reference nulliparous) |  |  |  |  |  |  |  |  |  |
| Parous, no previous PE | -0.02247 | 0.00615 | <0.001 |  |  |  |  |  |  |
| Parous, previous PE | -0.01985 | 0.01349 | 0.141 |  |  |  |  |  |  |

**Table S7** Number of cases per study group included in analysis at each clinical visit.

| Visit | Group 1 | Group 2 | Group 3 | Group 4 |
| --- | --- | --- | --- | --- |
| 1 | 407 | 598 | 29 | 44 |
| 2 | 401 | 586 | 29 | 44 |
| 3 | 356 | 530 | 26 | 20 |

**Table S8** Sensitivity analysis of mixed-effects models for hemodynamic variables.

| **Parameter** | **Log_10_ Heart rate** | | | **Log_10_ Stroke volume** | | | **Log_10_ Cardiac output** | | |
| --- | --- | --- | --- | --- | --- | --- | --- | --- | --- |
| Fixed part | Estimate | Standard error | p-value | Estimate | Standard error | p-value | Estimate | Standard error | p-value |
| Intercept | 1.88200 | 0.02209 | <0.001 | 1.28100 | 0.07872 | <0.001 | 0.37210 | 0.08405 | <0.001 |
| Group (reference: group 1) |  |  |  |  |  |  |  |  |  |
| Group 2 | -0.00397 | 0.02711 | 0.884 | 0.02559 | 0.04528 | 0.026 | 0.02472 | 0.04878 | 0.612 |
| Group 3 | -0.17440 | 0.09216 | 0.059 | -0.05673 | 0.15370 | -0.057 | -0.22730 | 0.16580 | 0.171 |
| Group 4 | 0.04338 | 0.04389 | 0.323 | -0.08578 | 0.07338 | -0.086 | -0.04900 | 0.07923 | 0.536 |
| Visit (reference visit 1) |  |  |  |  |  |  |  |  |  |
| Visit 2 | 0.01223 | 0.01496 | 0.414 | 0.02291 | 0.02750 | 0.405 | 0.03463 | 0.03119 | 0.267 |
| Visit 3 | 0.02415 | 0.03841 | 0.530 | 0.18680 | 0.06738 | 0.006 | 0.19670 | 0.07490 | 0.009 |
| Interaction groups with Clinical visit |  |  |  |  |  |  |  |  |  |
| Group 2 * Visit 2 | -0.00166 | 0.00365 | 0.649 | -0.00195 | 0.00671 | 0.771 | -0.00343 | 0.00763 | 0.653 |
| Group 3 * Visit 2 | 0.01241 | 0.01086 | 0.253 | 0.00483 | 0.01997 | 0.809 | 0.01757 | 0.02269 | 0.439 |
| Group 4 * Visit 2 | 0.01029 | 0.00921 | 0.264 | 0.01974 | 0.01695 | 0.244 | 0.03025 | 0.01925 | 0.116 |
| Group 2 * Visit 3 | 0.00307 | 0.00416 | 0.462 | 0.01463 | 0.00739 | 0.048 | 0.01792 | 0.00843 | 0.034 |
| Group 3 * Visit 3 | -0.00209 | 0.01239 | 0.866 | 0.02341 | 0.02198 | 0.287 | 0.02127 | 0.02506 | 0.396 |
| Group 4 * Visit 3 | -0.04109 | 0.01313 | 0.002 | 0.05611 | 0.02342 | 0.017 | 0.01599 | 0.02669 | 0.549 |
| Height (cm) |  |  |  | 0.00250 | 0.00045 | <0.001 | 0.00101 | 0.00048 | 0.036 |
| Weight (kg) |  |  |  | 0.00119 | 0.00023 | <0.001 | 0.00166 | 0.00025 | <0.001 |
| Parity (reference nulliparous) |  |  |  |  |  |  |  |  |  |
| Parous, no previous PE |  |  |  | 0.01557 | 0.00562 | 0.006 | 0.02304 | 0.00597 | <0.001 |
| Parous, previous PE |  |  |  | 0.02078 | 0.01171 | 0.076 | 0.02995 | 0.01244 | 0.016 |
| Chronic hypertension | 0.02131 | 0.00883 | 0.016 | 0.03687 | 0.01487 | 0.013 | 0.05419 | 0.01580 | 0.001 |
| Number of visits (reference: two visits) | -0.00508 | 0.00766 | 0.507 | 0.02791 | 0.01287 | 0.030 | 0.02305 | 0.01401 | 0.100 |
| Interaction study group with number of visits |  |  |  |  |  |  |  |  |  |
| Group 2 * three visits | 0.00520 | 0.00938 | 0.579 | -0.02548 | 0.01564 | 0.104 | -0.02223 | 0.01684 | 0.187 |
| Group 3 * three visits | 0.06232 | 0.03160 | 0.049 | 0.00066 | 0.05268 | 0.990 | 0.06087 | 0.05682 | 0.284 |
| Group 4 * three visits | -0.01846 | 0.01690 | 0.275 | -0.00665 | 0.02832 | 0.815 | -0.02458 | 0.03054 | 0.421 |
| Interaction clinical visit with number of visits |  |  |  |  |  |  |  |  |  |
| Visit 2 * three visits | -0.00005 | 0.00512 | 0.992 | -0.00335 | 0.00941 | 0.722 | -0.00374 | 0.01067 | 0.726 |
| Visit 3 * three visits | -0.00150 | 0.01286 | 0.907 | -0.06544 | 0.02252 | 0.004 | -0.06357 | 0.02504 | 0.011 |
| **Parameter** | **Log_10_ Systemic vascular resistance** | | | **Log_10_ Mean arterial pressure** | | |  |  |  |
| Fixed part | Estimate | Standard error | p-value | Estimate | Standard error | p-value |  |  |  |
| Intercept | 3.39600 | 0.08543 | <0.001 | 1.85300 | 0.03216 | <0.001 |  |  |  |
| Group (reference: group 1) |  |  |  |  |  |  |  |  |  |
| Group 2 | 0.00112 | 0.04980 | 0.982 | 0.02519 | 0.01850 | 0.174 |  |  |  |
| Group 3 | 0.19410 | 0.16910 | 0.251 | -0.03404 | 0.06320 | 0.590 |  |  |  |
| Group 4 | 0.14650 | 0.08055 | 0.069 | 0.10530 | 0.03008 | 0.000 |  |  |  |
| Visit (reference visit 1) |  |  |  |  |  |  |  |  |  |
| Visit 2 | -0.03421 | 0.03218 | 0.288 | 0.00080 | 0.00980 | 0.935 |  |  |  |
| Visit 3 | -0.15930 | 0.07812 | 0.042 | 0.03895 | 0.02594 | 0.133 |  |  |  |
| Interaction groups with time |  |  |  |  |  |  |  |  |  |
| Group 2 * Visit 2 | 0.00028 | 0.00787 | 0.972 | -0.00323 | 0.00239 | 0.177 |  |  |  |
| Group 3 * Visit 2 | -0.01618 | 0.02342 | 0.490 | 0.00130 | 0.00712 | 0.855 |  |  |  |
| Group 4 * Visit 2 | -0.02736 | 0.01987 | 0.169 | 0.00277 | 0.00604 | 0.646 |  |  |  |
| Group 2 * Visit 3 | -0.02293 | 0.00877 | 0.009 | -0.00504 | 0.00288 | 0.080 |  |  |  |
| Group 3 * Visit 3 | -0.01303 | 0.02610 | 0.618 | 0.00805 | 0.00857 | 0.348 |  |  |  |
| Group 4 * Visit 3 | 0.00974 | 0.02776 | 0.726 | 0.02614 | 0.00901 | 0.004 |  |  |  |
| Height (cm) | -0.00148 | 0.00047 | 0.002 | -0.00037 | 0.00018 | 0.045 |  |  |  |
| Weight (kg) |  |  |  | 0.00159 | 0.00009 | <0.001 |  |  |  |
| Smoking (reference non-smokers) |  |  |  | -0.01195 | 0.00396 | 0.003 |  |  |  |
| Parity (reference nulliparous) |  |  |  |  |  |  |  |  |  |
| Parous, no previous PE | -0.02180 | 0.00608 | <0.001 |  |  |  |  |  |  |
| Parous, previous PE | -0.02284 | 0.01266 | 0.071 |  |  |  |  |  |  |
| Chronic hypertension |  |  |  | 0.03784 | 0.00604 | 0.000 |  |  |  |
| Number of visits (reference: two visits) | -0.02180 | 0.01431 | 0.128 | 0.00146 | 0.00526 | 0.782 |  |  |  |
| Interaction study group with number of visits |  |  |  |  |  |  |  |  |  |
| Group 2 * three visits | 0.02594 | 0.01720 | 0.132 | 0.00451 | 0.00640 | 0.481 |  |  |  |
| Group 3 * three visits | -0.03229 | 0.05795 | 0.577 | 0.02971 | 0.02167 | 0.171 |  |  |  |
| Group 4 * three visits | 0.00950 | 0.03112 | 0.760 | -0.01619 | 0.01157 | 0.162 |  |  |  |
| Interaction clinical visit with number of visits |  |  |  |  |  |  |  |  |  |
| Visit 2 * three visits | -0.00199 | 0.01101 | 0.857 | -0.00577 | 0.00335 | 0.086 |  |  |  |
| Visit 3 * three visits | 0.04786 | 0.02615 | 0.068 | -0.01602 | 0.00867 | 0.065 |  |  |  |
